# Liver Function Biomarkers and Lung Cancer Risk: A Prospective Cohort Study in the UK Biobank

**DOI:** 10.1111/crj.70042

**Published:** 2024-12-25

**Authors:** Xiangyu Sun, Zeqin Guo, Yanpei Zhang, Zhuangzhuang Liu, Jingrong Xiong, Mingliang Cai, Jiale Tan, Yan Lin, Zihang Yu, Kunheng Du, Enli Lu, Xiaolin Xia

**Affiliations:** ^1^ Department of Radiation Oncology, Nanfang Hospital Southern Medical University Guangzhou China; ^2^ Zhuhai People's Hospital Jinan University Zhuhai China; ^3^ Department of Radiation Oncology Yunfu People's Hospital Yunfu China

**Keywords:** liver function biomarkers, lung cancer, Mendelian randomization, risk prediction, smoking

## Abstract

**Background:**

As the primary organ of metabolism and detoxification, the liver may contribute to the pathogenesis of lung cancer. We aimed to illuminate the intricate link between liver function biomarkers and lung cancer risk, as well as delineate the role of smoking behavior within this association.

**Methods:**

We investigated the associations of seven liver function biomarkers levels (alkaline phosphatase [ALP], alanine transaminase [ALT], total bilirubin [TBIL], albumin [ALB], gamma‐glutamyltransferase [GGT], aspartate transaminase [AST], and total protein [TP]) with lung cancer risk across the UK Biobank (*N* = 337 499) through restricted cubic splines and Cox proportional hazards models. Moreover, Mendelian randomization (MR) was utilized to evaluate the causal effect of smoking behavior on these biomarkers. Then a lung cancer risk prediction model was developed among smokers by backward stepwise logistic regression.

**Results:**

During a median follow‐up of 13.3 years, 3003 lung cancer cases were identified. We found ALP levels positively associated with lung cancer risk, whereas ALT, TBIL, ALB, and AST were inversely correlated; TP exhibited a U‐shaped association, whereas GGT displayed a mirrored J‐shaped relationship. These associations were amplified among smokers. MR analysis indicated that smoking behavior could increase ALP (odds ratio [OR]: 1.05) and GGT (OR: 1.15) levels while decreasing TBIL (OR: 0.92), ALB (OR: 0.92), and TP (OR: 0.96) levels. The lung cancer risk model incorporating these biomarkers in smokers demonstrated robust discrimination.

**Conclusion:**

Our finding provides perspectives and evidences towards the intricate crosstalk between the hepatic and pulmonary systems, as well as the processes through which tobacco catalyzes lung carcinogenesis.

## Graphical abstract

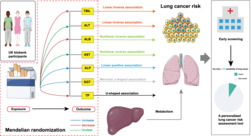

## 1 Introduction

Lung cancer remains one of the most common and deadly disease in the United States, with an estimate of 1 958 310 new diagnosed cases and 609 820 deaths in 2023 [1]. Smoking is thought to be the leading cause of lung cancer, but it does not imply an inevitable diagnosis [2]. Further elucidating the specific mechanisms and identifying risk factors of lung cancer in smokers are of great importance. Previous studies have shown that smoking not only damages the lungs by releasing harmful substances but also impairs systemic metabolism and detoxication [3], which play an important role in the development of lung cancer. For example, tumor cells derive energy through metabolic pathways, and metabolites can modulate signalling and gene expression to promote tumor proliferation and invasion [4].

As the primary site of metabolism and detoxification, the liver maintains homeostasis by synthesizing, storing, metabolizing, and clearing some substances in the body [5]. It plays a key role in the degradation and elimination of tobacco toxin. Liver function biomarkers such as alanine transaminase (ALT), aspartate transaminase (AST), total bilirubin (TBIL), gamma‐glutamyltransferase (GGT), alkaline phosphatase (ALP), total protein (TP), and albumin (ALB) [6], constitute a panel of laboratory tests indicating hepatic metabolic functions [7]. These markers also reflect the relationship between liver and other diseases in metabolism [8].

However, few studies have investigated the associations between liver function biomarkers and lung cancer risk. Furthermore, whether those associations differed in smokers versus nonsmokers and whether smoking behavior has a causal effect on these biomarkers have yet to be elucidated. To address these questions, we performed a prospective cohort study in the UK Biobank (UKB). We systematically evaluated whether and how these liver function biomarkers and lung cancer risk are related and found an interaction between smoking and liver function biomarkers in subgroup analysis. We further used Mendelian randomization (MR) analysis with single nucleotide polymorphisms (SNPs) as instruments to confirm the causal effect of smoking on these biomarkers. Finally, a lung cancer risk prediction model was developed based on liver function biomarkers and other risk factors in smokers.

## 2 Materials and Methods

### 2.1 Study Population

The UKB is a prospective cohort study that recruited more than 500 000 participants (aged 37–73 years) in 22 assessment centers throughout the United Kingdom between 2006 and 2010 [9]. Follow‐up spanning over a decade was conducted. At the baseline recruitment visit, participants completed touchscreen questionnaires regarding their sociodemographics, health and medical history, lifestyle, and environment. Then they underwent a physical assessment including height, waist, body weight, blood pressure, and lung function. The study protocol received ethical approval from the North West Multi‐centre Research Ethics Committee (reference 10/NW/0157), and all the participants provided their consent for long‐term follow‐up. Detailed description of the UKB design is available online (www.ukbiobank.ac.uk). Of the initial 502 401 UKB participants, those with a prebaseline diagnosis of cancer (*n* = 40 727), missing data on any of the seven liver function markers (*n* = 68 472) or some covariate data (*n* = 2543) were excluded. In the follow‐up, we excluded those who died (*n* = 23 760) or lost to follow‐up (*n* = 911) before lung cancer was diagnosed and those who were indicated as outliers (*n* = 28 140) based on a box–whisker plot [10]. Eventually, a total of 337 499 participants were included in our analysis (see the flowchart in Figure S1).

### 2.2 Biomarker Measurements

Blood samples were collected at both the initial recruitment from all participants and a follow‐up visit between 2012 and 2013 from a subset of 18 000 participants, and seven indicators related to liver function (ALT, AST, TBIL, GGT, ALP, TP, TBIL, and ALB) were incorporated into our study. These samples were collected and transported overnight in standard vacutainers to a central laboratory where they were processed. Then aliquots of blood constituent were stored in −80°C archives [11]. ALP, ALT, GGT, and AST were analyzed using enzymatic rate method with Beckman Coulter AU5800, whereas ALB, TP, and TBIL using the colorimetric method. The average coefficient of variation (CV) of quality control samples in the laboratory was controlled in a low range (Table S1) [6]. Participants with extreme outliers for any of these seven biomarkers (*n* = 28 140) were excluded due to being indicated as extreme outliers by a box–whisker plot [10], so the circulating levels of liver function biomarkers were largely within the normal range (Table S2). Normal range is based on Beckman Coulter AU5800 determined reference range for circulating liver function markers(https://www.beckmancoulter.com/support/tech‐docs).

### 2.3 Outcome Ascertainment

Cancer incidence and death cases within the UKB were conducted through linkage to the National Cancer and National Death Registration offices. Participants were followed up from the date of baseline attendance until the date of diagnosis of lung cancer or the end of the study period (1 May 2022), whichever occurred first. Lung cancer was defined by 10th Revision of the International Classification of Diseases (ICD‐10) based on the site of lung cancer: main bronchus (C34.0), upper lobe bronchus or lung (C34.1), middle lobe bronchus or lung (C34.2), lower lobe, bronchus or lung (C34.3), overlapping lesion of bronchus and lung (C34.8), and bronchus or lung unspecified (C34.9).

### 2.4 MR Analysis

Genetic IVs for smoking were extracted from the GWAS & Sequencing Consortium of Alcohol and Nicotine use (GSCAN) [12]. These data were obtained from a European population of 607 291 participants. Details are showed in Table S3. Data for the genome‐wide association study of liver function biomarkers were derived from published studies from the UKB (Table S4).

MR analyses were performed using R packages “TwoSampleMR” with smoking initiation (the predisposition to ever being a regular smoker as opposed to never initiating smoking) as exposure variables and liver function biomarkers as outcome variables.

For selection instrumental variables (IVs), standard parameters were utilized (10 000 kb aggregation window, *r*2 cutoff 0.001) to eliminate variants in linkage disequilibrium (LD). In total, 93 SNPs associated with smoking initiation at Stringent thresholds (*p* < 5 × 10^−8^) [13]. We used the *F* statistic to evaluate the IV strength so as to circumvent potential weak instrumental bias (F=(n−2) ×$\left(\frac{R^{2}}{1-R^{2}}\right)$, *R* ^2^ = 2 × (1 − EAF) × EAF × β^2^; *R* ^2^ is the proportion of the variability of the smoking initiation explained by each instrument, EAF is effect allele frequency, beta is the estimated genetic effect, and *n* is the sample size) [14]. *F* > 10 indicated a sufficiently robust correlation between the IV and exposure. The inverse variance weighted (IVW) approach was utilized as the primary analysis, and two additional multiple regression methods, MR‐Egger and the weighted median, were employed for sensitivity analyses. Then we applied MR‐Egger regression tests to evaluate potential horizontal pleiotropy [15].

### 2.5 Statistical Analysis

Baseline characteristics of participants were described across lung cancer status. Continuous variables are presented as means ± SD, and categorical variables as proportions.

Nonlinear associations between these liver function biomarkers and lung cancer risk were explored using restricted cubic splines (RCS) using R packages “Rcssci” [16]. The knots between 3 and 7 were selected as the lowest value for the Akaike information criterion [17]. The reference value (OR = 1) was set at the 10th percentile or the inflection point, depending on the shape of the figure.

We calculated hazard ratios (HRs) and 95% confidence intervals (CIs) using two Cox proportional hazards regression models that included an increasing number of covariates: Model 1 was adjusted for age, sex, ethnic, and Townsend deprivation index. Model 2 was further adjusted for a set of potential confounders, including weight, BMI, waistline, smoking status, alcohol drinker status, process meat, diabetes, asthma, hayfever and/or allergic rhinitis and/or eczema, emphysema and/or bronchitis, and forced expiratory volume in 1 s (FEV1). Schoenfeld residuals was used to test the proportional hazards assumption [18]. The liver function biomarkers were further divided into five categories, taking into account the normal reference range, data distribution, and easy‐to‐understand numbers. And if linear correlation was found in RCS, we standardized (Z‐score) the levels of this biomarker to investigate the impact of per SD increase on lung cancer.

The following sensitivity analyses were performed: (1) excluding incident cases occurred in the first 2 years of follow‐up (*n* = 332) and (2) excluding participants with hepatitis and other liver/hepatobiliary disease at recruitment (*n* = 803) using the Cox proportional hazards model. Subgroup analysis was performed to explore whether the association between liver function biomarkers and lung cancer risk is somehow dependent on demographic factors, particularly smoking status [19].

To evaluate the reproducibility of these liver function biomarkers among participants with repeated measurements, we calculated intraclass correlation coefficients (ICCs) after excluding participants diagnosed with lung cancer during the two measurement periods (*n* = 76) [6].

For the development of our risk prediction model in smokers, the known risk factors of lung cancer that are routinely available or can be easily ascertained during a standard consultation were selected as candidate variables for backward stepwise regression selection. These variables included age, sex, FEV1, waistline, family history of lung cancer, hayfever and/or allergic rhinitis and/or eczema, emphysema and/or bronchitis, and smoking pack‐years, together with seven liver function biomarkers. Missing values of smoking pack‐years were imputed using multiple imputation (MI) method, taking the average 20 imputations [20]. Models were developed based on the results of backward stepwise logistic regression [21].

Concordance index (C‐index) was used to assess the discrimination ability of the model. The net reclassification improvement (NRI) [22] and integrated discrimination improvement (IDI) [23] were also used to evaluate the added predictive ability of liver function markers in the lung cancer risk prediction model. High NRI and IDI score means the new model has better discrimination [24]. Hosmer–Lemeshow test was used to assess the calibration of the model.

Analyses were conducted using SPSS (Version 26) and R (Version 4.2.3) with packages survival, irr, rms, rcssci, nricens, and PredictABEL.

## 3 Results

### 3.1 Baseline Characteristics

Of all 337 499 participants eventually included in the study, 3003 incident lung cancer cases were identified during a median follow‐up of 13.3 years (incidence rate: 6.75 events per 10 000 person‐years). Participants with lung cancer were typically older and smokers and more likely to be male and obese. This lung cancer cohort is also likely to have a family history of lung cancer, a history of emphysema and/or bronchitis, diabetes, and worse lung function (FEV1) but less likely to have the history of hayfever and/or allergic rhinitis and/or eczema. Surprisingly, we found participants with lung cancer tend to have higher concentrations of ALP and GGT and lower concentrations of ALB, ALT, AST, TBIL and TP in the blood (Table 1).

**TABLE 1:** Demographic and clinical characteristics of participants.

Characteristic	Participants with lung cancer	Participants without lung cancer	Overall
	(*N* = 3003)	(*N* = 334 496)	(*N* = 337 499)
Sex			
Male	1539 (51.2%)	147 541 (44.1%)	149 080 (44.2%)
Female	1464 (48.8%)	186 955 (55.9%)	188 419 (55.8%)
Age (years)			
< 50	115 (3.8%)	85 197 (25.5%)	85 312 (25.3%)
50–55	226 (7.5%)	53 405 (16.0%)	53 631 (15.9%)
55–60	499 (16.6%)	61 566 (18.4%)	62 065 (18.4%)
60–65	1023 (34.1%)	78 495 (23.5%)	79 518 (23.6%)
> 65	1140 (38.0%)	55 833 (16.7%)	56 973 (16.9%)
Ethnic			
White	2753 (91.7%)	302 705 (90.5%)	305 458 (90.5%)
Mixed	125 (4.2%)	12 379 (3.7%)	12 504 (3.7%)
Asian or Asian British	86 (2.9%)	11 938 (3.6%)	12 024 (3.6%)
Black or Black British	11 (0.4%)	2034 (0.6%)	2045 (0.6%)
Chinese	6 (0.2%)	1116 (0.3%)	1122 (0.3%)
Other ethnic group	16 (0.5%)	3122 (0.9%)	3138 (0.9%)
Unknown	6 (0.2%)	1202 (0.4%)	1208 (0.4%)
Weight (kg)			
Mean (SD)	77.5 (15.7)	77.5 (15.6)	77.5 (15.6)
Median [Min, Max]	76.1 [36.0, 155]	75.9 [30.1, 195]	75.9 [30.1, 195]
BMI (kg/m^2^)			
Mean (SD)	27.4 (4.72)	27.3 (4.68)	27.3 (4.69)
Median [Min, Max]	26.8 [15.9, 52.5]	26.6 [12.1, 68.1]	26.6 [12.1, 68.1]
Waistline (cm)			
Mean (SD)	92.6 (13.2)	89.6 (13.2)	89.6 (13.2)
Median [Min, Max]	92.0 [56.0, 148]	89.0 [20.0, 197]	89.0 [20.0, 197]
Systolic blood pressure (mmHg)			
Mean (SD)	143 (20.2)	139 (19.4)	139 (19.5)
Median [Min, Max]	142 [89.0, 224]	137 [62.0, 268]	137 [62.0, 268]
Missing	276 (9.2%)	21 139 (6.3%)	21 415 (6.3%)
Alcohol drinker status			
Never	110 (3.7%)	14 744 (4.4%)	14 854 (4.4%)
Previous	225 (7.5%)	11 048 (3.3%)	11 273 (3.3%)
Current	2664 (88.7%)	308 254 (92.2%)	310 918 (92.1%)
Unknown	4 (0.1%)	450 (0.1%)	454 (0.1%)
Smoking status			
Never	413 (13.8%)	188 567 (56.4%)	188 980 (56.0%)
Previous	1330 (44.3%)	111 706 (33.4%)	113 036 (33.5%)
Current	1230 (41.0%)	33 039 (9.9%)	34 269 (10.2%)
Unknown	30 (1.0%)	1184 (0.4%)	1214 (0.4%)
Process meat			
Never	232 (7.7%)	32 061 (9.6%)	32 293 (9.6%)
Less than once a week	774 (25.8%)	103 309 (30.9%)	104 083 (30.8%)
Once a week	865 (28.8%)	97 055 (29.0%)	97 920 (29.0%)
2–4 times a week	929 (30.9%)	88 525 (26.5%)	89 454 (26.5%)
5–6 times a week	147 (4.9%)	10 168 (3.0%)	10 315 (3.1%)
Once or more daily	49 (1.6%)	2570 (0.8%)	2619 (0.8%)
Unknown	7 (0.2%)	808 (0.2%)	815 (0.2%)
Townsend deprivation index			
Mean (SD)	−0.114 (3.59)	−1.38 (3.04)	−1.36 (3.05)
Median [Min, Max]	−0.857 [−6.16, 10.9]	−2.19 [−6.26, 11.0]	−2.19 [−6.26, 11.0]
FEV1 (L)			
< 3	2081 (69.3%)	189 955 (56.8%)	192 036 (56.9%)
3–4	433 (14.4%)	91 031 (27.2%)	91 464 (27.1%)
> 4	49 (1.6%)	27 372 (8.2%)	27 421 (8.1%)
Unknown	440 (14.7%)	26 138 (7.8%)	26 578 (7.9%)
History of diabetes			
No	2744 (91.4%)	319 131 (95.4%)	321 875 (95.4%)
Yes	243 (8.1%)	14 350 (4.3%)	14 593 (4.3%)
Unknown	16 (0.5%)	1015 (0.3%)	1031 (0.3%)
History of emphyse and/or machronic and/or bronchitis			
No	2742 (91.3%)	330 155 (98.7%)	332 897 (98.6%)
Yes	261 (8.7%)	4341 (1.3%)	4602 (1.4%)
History of asthma			
No	2638 (87.8%)	296 235 (88.6%)	298 873 (88.6%)
Yes	365 (12.2%)	38 261 (11.4%)	38 626 (11.4%)
History of hay fever, allergic rhinitis, or eczema			
No	2569 (85.5%)	256 258 (76.6%)	258 827 (76.7%)
Yes	434 (14.5%)	78 238 (23.4%)	78 672 (23.3%)
Family history of lung cancer			
No	2395 (79.8%)	291 442 (87.1%)	293 837 (87.1%)
Yes	487 (16.2%)	33 299 (10.0%)	33 786 (10.0%)
Unknown	121 (4.0%)	9755 (2.9%)	9876 (2.9%)
ALB (g/L)			
Mean (SD)	44.5 (2.61)	45.2 (2.57)	45.2 (2.57)
Median [Min, Max]	44.5 [33.7, 55.9]	45.2 [33.4, 56.9]	45.2 [33.4, 56.9]
ALP (U/L)			
Mean (SD)	89.8 (22.9)	81.6 (21.6)	81.6 (21.6)
Median [Min, Max]	87.5 [20.9, 180]	79.2 [8.00, 180]	79.3 [8.00, 180]
ALT (U/L)			
Mean (SD)	20.8 (8.47)	21.7 (9.20)	21.6 (9.20)
Median [Min, Max]	19.1 [3.49, 59.5]	19.6 [3.01, 59.6]	19.6 [3.01, 59.6]
GGT (U/L)			
Mean (SD)	34.7 (19.0)	30.6 (17.9)	30.6 (17.9)
Median [Min, Max]	29.1 [6.40, 105]	25.1 [5.00, 106]	25.1 [5.00, 106]
TBIL (μmol/L)			
Mean (SD)	8.15 (3.07)	8.72 (3.33)	8.71 (3.32)
Median [Min, Max]	7.51 [2.67, 22.2]	7.99 [1.08, 22.3]	7.98 [1.08, 22.3]
TP (g/L)			
Mean (SD)	72.1 (4.25)	72.5 (4.01)	72.5 (4.01)
Median [Min, Max]	71.9 [59.0, 88.9]	72.3 [54.7, 90.7]	72.3 [54.7, 90.7]
AST (U/L)			
Mean (SD)	24.4 (5.72)	24.9 (5.87)	24.8 (5.87)
Median [Min, Max]	23.6 [8.80, 50.3]	24.0 [3.30, 50.7]	24.0 [3.30, 50.7]

Abbreviations: ALB, albumin; ALP, alkaline phosphatase; ALT, alanine transaminase; ALT, alanine transaminase; AST, aspartate transaminase; GGT, gamma‐glutamyltransferase; TP, total protein.

### 3.2 Observational Associations Between Circulating Biomarkers of Liver Function and Lung cancer Risk

**FIGURE 1:**
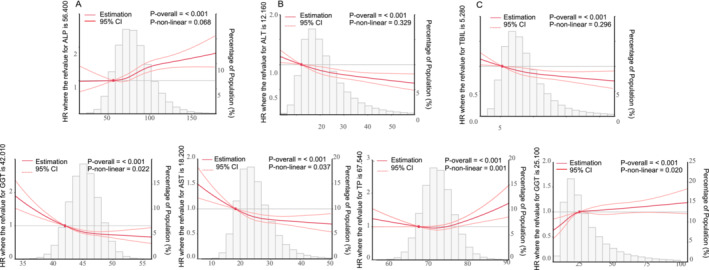
The restricted cubic spline for the associations between levels of liver function biomarkers and the risk of lung cancer. The cloud represents the 95% confidence intervals of the hazard ratio. The lines represent hazard ratios (HRs, solid lines) and 95% confidence intervals (CIs, long dashed lines) after multivariable adjustment for age (< 50 years, 50–55 years, 55–60 years, 60–65 years, > 65 years), sex (male, female), ethnic (white, mixed, Asian or Asian British, Black or Black British, Chinese, Other ethnic group, unknown), Townsend deprivation index (quartile), weight, BMI, waistline, smoking status (never, previous, current, unknown), alcohol drinker status (never, previous, current, unknown), process meat(never, less than once a week, once a week, 2–4 times a week, 5–6 times a week, once or more daily, unknown), diabetes (no, yes, unknown), asthma (no, yes), hayfever and/or allergic rhinitis and/or eczema (no, yes), emphysema and/or bronchitis (no, yes), family history of lung cancer (no, yes), and FEV1(< 3 L, 3–4 L, > 4 L). The histograms represent the distribution of concentrations of circulating liver function markers. ALB, albumin; ALP, alkaline phosphatase; ALT, alanine transaminase; ALT, alanine transaminase; AST, aspartate transaminase; CI, confidence interval; FEV1, forced expiratory volume in 1 s; GGT, gamma‐glutamyltransferase; HR, hazard ratio.TP, total protein.

To investigate the association between lung cancer risk and hepatic function biomarkers, we utilized restricted cubic spline analysis and two Cox regression models adjusting all potential covariates (Figure 1 and Table 2). In general, the ALP levels exhibited a significant and positive correlation with lung cancer risk. Participants were stratified into five groups based on circulating ALP levels. The multivariable HR (95% CI) in group 55–75 U/L, 75–95 U/L, 95–115 U/L, and > 115 U/L were 1.06 (0.87–1.28), 1.24 (1.03–1.50), 1.57 (1.30–1.91), and 1.54 (1.25–1.89), respectively, compared with the reference group < 55 U/L (Table 2). Circulating levels of ALT, TBIL, ALB, and AST were inversely associated with lung cancer risk [Model 2: HR (95% CI) = 1 (Ref), 0.89 (0.73–1.08), 0.81 (0.66–0.99), 0.73 (0.58–0.92), and 0.67 (0.51–0.88) for ALT groups; HR (95% CI) = 1 (Ref), 0.88 (0.78–1.00), 0.83 (0.73–0.96), 0.83 (0.71–0.97), and 0.72 (0.60–0.85) for the TBIL groups; HRs (95% CI) = 1 (Ref), 0.85 (0.70–1.04), 0.71 (0.58–0.86), 0.65 (0.53–0.79), and 0.55 (0.43–0.72) for ALB groups; HR (95% CI) = 1 (Ref), 0.67 (0.51–0.89), 0.60 (0.45–0.79), 0.51 (0.37–0.71), and 0.69 (0.42–1.14) for AST groups (Figure 1 and Table 2)]. A mirrored J‐shaped association was observed between circulating GGT levels and the risk of lung cancer. As the concentration increasing, the HR initially increased then plateaued at nonsignificance, exhibiting solely a subtle escalating tendency (Figure 1G and Table 2). The 25–35 U/L group including the inflection point was set as the reference group (Table 2). As for TP, a U‐shaped relationship was discerned. The lowest HR for lung cancer was found in the 68–74 g/L group, which was set as the reference group. The HR in the ≥ 80 group was 1.30 (95% CI: 1.08–1.57). However, the HRs were not significant for lower level groups and 74–80 group, with only a slightly increasing trend noted (Figure 1F and Table 2). Besides, restricted cubic spline analysis demonstrated a linear relationship between the levels of ALP, ALT, TBIL, and lung cancer risk (*p*‐nonlinear = 0.068, 0.329, 0.296, respectively; Figure 1A–C). The multivariable‐adjusted HR (per 1‐SD increment) (95% CI, Model 2) was 1.15 (1.11–1.19) for ALP, 0.90 (0.86–0.94) for GGT, and 0.92 (0.88–0.96) for TBIL (Table 2).

**TABLE 2:** The hazard risk (HR) with 95% confidence intervals (95% CI) between different liver function biomarkers and lung cancer.

ALP (U/L)	< 55	55–75	75–95	95–115	> 115	Per‐SD increase
Model 1	1 (Ref)	1.11 (0.91–1.34)	1.45 (1.21–1.75)	2.05 (1.70–2.49)	2.23 (1.82–2.74)	1.28 (1.24–1.32)
Model 2	1 (Ref)	1.0 6 (0.87–1.28)	1.24 (1.03–1.50)	1.57 (1.30–1.91)	1.54 (1.25–1.89)	1.15 (1.11–1.19)
ALT(U/L)	< 10	10–15	15–20	20–25	> 25	Per‐SD increase
Model 1	1 (Ref)	0.80 (0.66–0.97)	0.67 (0.55–0.82)	0.61 (0.49–0.76)	0.55 (0.42–0.72)	0.86 (0.82–0.89)
Model 2	1 (Ref)	0.89 (0.73–1.08)	0.81 (0.66–0.99)	0.73 (0.58–0.92)	0.67 (0.51–0.88)	0.90 (0.86–0.94)
TBIL (μmol/L)	< 5	5–7.5	7.5–10	10–12.5	> 12.5	Per‐SD increase
Model 1	1 (Ref)	0.71 (0.62–0.80)	0.56 (0.49–0.64)	0.50 (0.43–0.58)	0.40 (0.34–0.48)	0.78 (0.75–0.81)
Model 2	1 (Ref)	0.88 (0.78–1.00)	0.83 (0.73–0.96)	0.83 (0.71–0.97)	0.72 (0.60–0.85)	0.92 (0.88–0.96)
ALB(g/L)	< 40	40–43	43–46	46–49	> 49	
Model 1	1 (Ref)	0.77 (0.63–0.94)	0.60 (0.50–0.73)	0.52 (0.42–0.63)	0.44 (0.34–0.56)	
Model 2	1 (Ref)	0.85 (0.70–1.04)	0.71 (0.58–0.86)	0.65 (0.53–0.79)	0.55 (0.43–0.72)	
GGT(U/L)	< 14	14–22	22–30	30–38	> 38	
Model 1	0.66 (0.56–0.77)	0.83 (0.75–0.91)	1 (Ref)	1.00 (0.89–1.13)	1.15 (1.03–1.27)	
Model 2	0.80 (0.68–0.95)	0.90 (0.81–0.99)	1 (Ref)	0.97 (0.86–1.09)	1.05 (0.94–1.16)	
AST(U/L)	< 15	15–25	25–35	35–45	> 45	
Model 1	1 (Ref)	0.50 (0.38–0.65)	0.37 (0.28–0.49)	0.31 (0.22–0.42)	0.43 (0.26–0.71)	
Model 2	1 (Ref)	0.67 (0.51–0.89)	0.60 (0.45–0.79)	0.51 (0.37–0.71)	0.69 (0.42–1.14)	
TP(g/L)	< 62	62–68	68–74	74–80	> 80	
Model 1	1.64 (0.99–2.73)	1.20 (1.08–1.33)	1 (Ref)	0.96 (0.88–1.04)	1.14 (0.94–1.37)	
Model 2	1.45 (0.87–2.41)	1.04 (0.94–1.15)	1 (Ref)	1.06 (0.97–1.15)	1.30 (1.08–1.57)	

*Note:* Model 1: Adjusted age, sex, ethnic, and Townsend deprivation index. Model 2: Adjusted model 1 + weight, BMI, waistline, smoking status, alcohol drinker status, process meat, diabetes, asthma, hayfever and/or allergic rhinitis and/or eczema, emphysema and/or bronchitis, family history of lung cancer, and forced expiratory volume in 1 s (FEV1).

Abbreviations: ALB, albumin; AST, aspartate transaminase; ALP, alkaline phosphatase; ALT, alanine transaminase; ALT, alanine transaminase; GGT, gamma‐glutamyltransferase; TP, total protein.

The levels of these biomarkers measured at two timepoints (4.4 years apart) in a subset of 18 000 participants were highly consistent (ICCs ranged between 0.62 and 0.85) (Table S5). This means that the circulating levels of liver function biomarkers among participants with repeated measurements were reproducible.

### 3.3 Sensitivity and Subgroup Analyses

We then performed sensitivity analyses to corroborate our findings. Results showed robust relationships after excluding the first 2 years of follow‐up as well as excluding participants with hepatitis and other liver/hepatobiliary disease at recruitment (Table S6).

Considering that age, sex, and smoking status have been regarded as strong risk factors for lung cancer, we performed subgroup analysis stratifying by them respectively. Clear pattern of interaction was observed between circulating levels of ALP, TBIL, ALB, GGT, and ever‐smoked (*p* of interaction = 0.003, 0.006, 0.014, 0.010, respectively) (Table S7). Among smokers, we found that lung cancer patients exhibited higher concentrations of ALP and GGT and lower levels of ALT, ALB, and AST compared to those without lung cancer (Figure S2), consistent with the patterns observed in Figure 1 and Table 2. However, these differences were attenuated among nonsmokers (Figure S3). In Cox regression model, much stronger links between these biomarkers and lung cancer risk were observed in smokers. Conversely, these associations were largely negligible among nonsmokers (Table S7). These evidence indicate that the relationship between these liver function indicators and lung cancer is primarily evident within the smoking population. Subgroup analysis stratifying by age (less than 60 years old vs. 60 years old or older) and sex were also performed. No heterogeneity was observed except GGT and TP in different age (*p* for interaction = 0.012, < 0.001, respectively; Tables S8 and S9).

### 3.4 MR Analysis

Given the interaction between smoking status and various liver function biomarkers, we subsequently examined the impact of smoking on these seven liver function markers. Separate sets of 85 genetically autonomous SNPs were independently chosen in each case to serve as instruments. Detailed information on the instruments is shown in Tables S10–S17.

**FIGURE 2:**
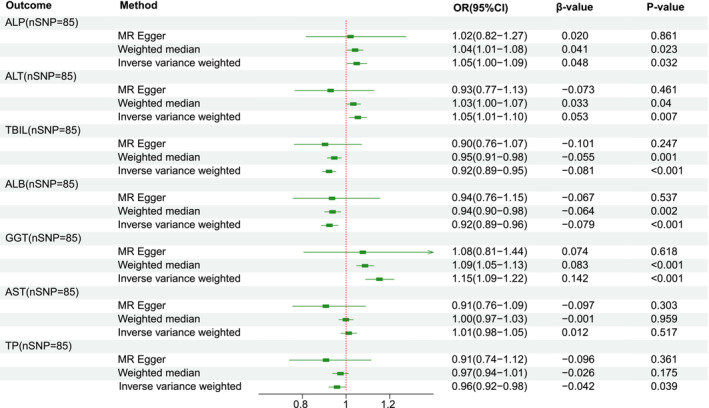
Odds ratio plot for smoking initiation and liver function markers in Mendelian randomization (MR) analysis. ALB, albumin; ALP, alkaline phosphatase; ALT, alanine transaminase; ALT, alanine transaminase; AST, aspartate transaminase; CI, confidence interval; GGT, gamma‐glutamyltransferase; OR, odds ratio; TP, total protein.

Using IVW method, we observed that smoking behavior significantly reduced the levels of TBIL, ALB, and TP, whereas increased the levels of ALP and GGT (Figure 2). These were all linked to elevated risks of lung cancer development in our previous analyses. As for AST, no significant association was observed. Although ALT showed a statistically significant result, the direction of beta value from MR‐Egger regression were incongruent with other methods, indicating this finding may lack requisite fidelity [25]. MR‐Egger analysis indicated there was no evidence of unbalanced horizontal pleiotropy (Table S18).

### 3.5 Circulating Levels of Liver Function Biomarkers and Lung Cancer Risk Prediction

Given the enhanced correlation between circulating biomarkers of hepatic function and risk of lung cancer in smokers, as well as the significant causal linkage between smoking and variations in concentrations of some hepatic biochemical markers, we further developed a lung cancer risk prediction model to evaluate the potential predictive value of these biomarkers for an early diagnosis of lung cancer in smokers.

Using backward stepwise logistic regression selection, two sets of models were generated: an epidemiological model including seven traditional predictors and a full model additionally incorporating these biomarkers (Table S19). To visualize our full model and facilitate its clinical implementation, we constructed a web‐based nomogram. By clicking on the URL (https://www.evidencio.com/models/show/10064?v=1.0) to enter the calculator interface, inputting personal data of the predictors, the individualized lung cancer risk probabilities can be quickly calculated (Figure S4).

The full model generated a C‐index of 0.813 (95% CI, 0.805–0.820). Significance was observed when compared with the epidemiological model (C‐index = 0.802, 95% CI, 0.794–0.810, *p* < 0.001; Table 3). Similarly, we found significant improvement of both category NRI (0.040, 95% CI, 0.034–0.077, *p* < 0.001) and continuous NRI (0.030, 95% CI, 0.017–0.047, *p* < 0.001), though the increase of IDI was quite small (0.0044, 95% CI, 0.0035–0.0053, *p* < 0.001) (Table 3). Hosmer–Lemeshow test showed the predicted and observed lung cancer risk probabilities agreed well, suggesting that the full model was well calibrated (*p* = 0.1631).

**TABLE 3:** Comparison of models with/without liver function biomarkers.

The epidemiological model	Value (95% CI)	
C‐index	0.802 (0.794–0.810)	

New model	Value (95% CI)	*p*
C‐index	0.813 (0.805–0.821)	< 0.001
NRI (categorical)	4.0% (3.4%–7.7%)	< 0.001
NRI (continuous)	3.0% (1.7%–4.7%)	< 0.001
IDI	0.44% (0.35%–0.53%)	< 0.001

Abbreviations: IDI, integrated discrimination improvement; NRI, net reclassification index.

## 4 Discussion

This large prospective cohort study yielded several notable findings. Firstly, circulating levels of ALP demonstrated a linear positive association with lung cancer risk, but ALT and TBIL exhibited linear inverse relationships; ALB and AST displayed nonlinear inverse correlations, whereas TP showed a U‐shaped association and GGT manifested a mirrored J‐shaped relationship. Secondly, the above trends were more pronounced in smokers, suggesting smokers as a key population for study. Thirdly, MR analysis indicated smoking may causally affect the concentrations of these biomarkers. We subsequently developed a lung cancer risk prediction model in smokers incorporating biomarkers of liver function that discriminated well between incident lung cancer cases and noncases, highlighting its clinical predictive value for early detection in high‐risk population.

The liver and the lungs are vital organs in human body, but few studies have directly investigated their relationship. Given the liver's pivotal role in metabolism and the metabolic involvement in lung cancer progression, it is reasonable to investigate the association between liver function and lung cancer risk. In the generally healthy population, fluctuations in biomarker levels may indicate decreased liver functionality [6], characterized by decreased synthetic and metabolic capacity as well as increased susceptibility to toxins [26], which may lead to other diseases. This may explain the trends between concentrations of these biomarkers and lung cancer.

Smokers are at high risk for lung cancer, as well as a focal population of our study that we discovered through subgroup analysis. Using MR methods, we further investigated the causal impacts of smoking behavior on liver function biomarkers, revealing that smoking elevates the levels of ALP and GGT while diminishing those of ALB, TP, and TBIL. These causal relationships are considered to associate with the increase of lung cancer risk in RCS and Cox regression models, which is logically coherent. We conjecture on the potential mechanisms of these causal relationships: Smoking may exacerbate the hepatic workload, resulting in cellular and tissue damage; This may cause hepatic enzymes such as ALP, GGT, and AST to be released into the bloodstream. Meanwhile, harmful substances in tobacco may impair proteins as well as suppress protein synthesis, leading to reduced serum TP and ALB levels. However, there is no causal link between AST and smoking, possibly because AST is more utilized to evaluate the extent of myocardial cell injury [27], and its concentration may be largely influenced by the heart; Regarding ALT, due to the discrepancy of β‐value direction obtained by MR‐egger and other methods, further research is required to elucidate its causal effects. Our study employed MR methods to assess the causal effects of smoking on liver function biomarkers, offering a novel insight into the intricate relationship between smoking and lung cancer.

Several lung cancer risk models have been developed in various populations, but each of them has their own limitations to a greater or lesser extent. Our study demonstrates the effectiveness of liver function biomarkers in lung cancer risk models, providing a useful tool for further screening in smokers. Of all the predictors included in our model, spirometry can be performed by medical personnel at hospitals, and circulating levels of liver function biomarkers can be obtained by biochemical examination [28]. Both are commonly used in clinical practice and easy to implement. Besides, other predictors can be easily obtained during a standard consultation, so the model has great potential to use in clinical practice on a wide scale.

The UKB database has its unique advantages. The large sample size allowed for a thorough investigation of the dose–response relationship between different liver function markers and the lung cancer risk as well as enabled the development of lung cancer risk prediction models. Due to the prospective nature of the UKB, we can directly model lung cancer incidence in one of the largest cohorts globally in a continuous manner in the UKB, and as all participants completed baseline assessments prior to lung cancer diagnoses, the possibility of differential reporting bias is minimized.

We also acknowledge several limitations of our research. Firstly, as an observational study, all residual confounding cannot be excluded [6]. Secondly, biomarkers and confounding variables were mainly measured at baseline, with repeated assessments for only a small subset of participants. Hence, for each patient, we are unable to investigate the impact of dynamic variations in the circulating levels of biomarkers on lung cancer over a certain period of time. However, we calculated ICCs to assess the reproducibility in circulating levels of liver function markers among participants with repeated measurements, which demonstrated strong reproducibility. Besides, although we found liver function biomarkers associated with lung cancer risk, whether there is a causal effect remains to be explored. Lastly, we have established a lung cancer risk prediction model among smokers, but there is a lack of valid evaluation for nonsmokers.

In conclusion, our study illuminates the intricate associations between liver function biomarkers and lung cancer risk while underscoring the integral role of tobacco in these relationships. MR analysis elucidated the influence of tobacco on liver biomarker levels. Subsequently, a personalized risk prediction model integrating liver biomarkers was developed in smokers. These findings forge metabolic connections between liver and lungs, furnish novel perspectives on smoking and liver function in lung carcinogenesis, and advocate promising applications of liver biomarkers for lung cancer screening and prevention efforts.

## Author Contributions

**Xiangyu Sun:** formal analysis, conceptualization, data curation, investigation, visualization, methodology, writing – original draft. **Zeqin Guo:** investigation, methodology, writing – review and editing. **Yanpei Zhang:** methodology, investigation.  **Zhuangzhuang Liu:** investigation, visualization. **Jingrong Xiong:** visualization. **Mingliang Cai:** Methodology. **Jiale Tan:** investigation, writing – review and editing. **Yan Lin:** visualization. **Zihang Yu:** investigation. **Kunheng Du:** investigation. **Enli Lu:** investigation. **Xiaolin Xia:** funding acquisition, writing – original draft, project administration.

## Ethics Statement

The UK Biobank study protocol received ethical approval from the North West Multi‐centre Research Ethics Committee (reference 21/NW/0157), and all the participants provided their consent for long‐term follow‐up.

## Conflicts of Interest

The authors declare no conflicts of interest.

## Supporting information


**Figure S1** Flowchart of participants’ inclusion in the UK Biobank.

**Figure S2** The liver enzymes of smokers with lung cancer compared with the liver enzymes of smokers without lung cancer.

**Figure S3** The liver enzymes of nonsmokers with lung cancer compared with the liver enzymes of nonsmokers without lung cancer.

**Figure S4** The appearance of the web‐based personalized lung cancer risk prediction model.

**Table S1** The average within‐laboratory coefficient of variation (CV) in quality‐control samples.
**Table S2.** Normal range for levels of liver function biomarkers and the percentage of samples within the normal range.
**Table S3.** Details for the GWAS information of exposure in MR analysis.
**Table S4.** Details for the GWAS information of outcome in MR analysis.
**Table S5.** Intraclass correlation coefficients of circulating liver function markers measured in repeated samples.
**Table S6.** The hazard ratios (HRs) with 95% confidence intervals (95% CI) between various liver function biomarkers and lung cancer in sensitivity analyses.
**Table S7.** The hazard risk (HR) with 95% confidence intervals (95% CI) between various liver function biomarkers and lung cancer by stratifying groups into smoker and nonsmoker using the full adjusted Cox proportional hazards model.
**Table S8.** The hazard ratios (HRs) with 95% confidence intervals (95% CIs) between various liver function biomarkers and lung cancer stratifying by age (less than 60 years old vs. 60 years old or older) using the full adjusted Cox proportional hazards model.
**Table S9.** The hazard ratios (HRs) with 95% confidence intervals (95% CIs) between various liver function biomarkers and lung cancer stratifying by sex using the full adjusted Cox proportional hazards model.
**Table S10.** 93 SNPs associated with smoking initiation.
**Table S11.** Detailed information on the SNPs instruments for the MR analysis of smoking initiation and alkaline phosphatase (ALP).
**Table S12.** Detailed information on the SNPs for the MR analysis of smoking initiation and alanine aminotransferase (ALT).
**Table S13.** Detailed information on the SNPs for the MR analysis of smoking initiation and total bilirubin (TBIL).
**Table S14.** Detailed information on the SNPs for the MR analysis of smoking initiation and albumin (ALB).
**Table S15.** Detailed information on the SNPs for the MR analysis of smoking initiation and aspartate aminotransferase (AST).
**Table S16.** Detailed information on the SNPs for the MR analysis of smoking initiation and gamma glutamyltransferase (GGT).
**Table S17.** Detailed information on the SNPs for the MR analysis of smoking initiation and total protein (TP).
**Table S18.** MR‐Egger analysis for testing potential horizontal pleiotropy.
**Table S19.** Odds ratios (ORs) with 95% confidence intervals (CIs) for the lung cancer risk models by backward stepwise logistic regression.

## Data Availability

The data that support the findings of this study are available from the UK Biobank, but restrictions apply to the availability of these data, which were used under license for the current study and so are not publicly available. Data are however available from the authors upon reasonable request and with permission of the UK Biobank (https://www.ukbiobank.ac.uk/enable‐your‐research/apply‐for‐access).
