# Supplementary figures and images for: Liver Function Biomarkers and Lung Cancer Risk: A Prospective Cohort Study in the UK Biobank
Source: Clin Respir J. 2024 Dec 25;18(12):e70042. doi: 10.1111/crj.70042 (PMC11669495; doi:10.1111/crj.70042)

**Supplementary Figure 1.**

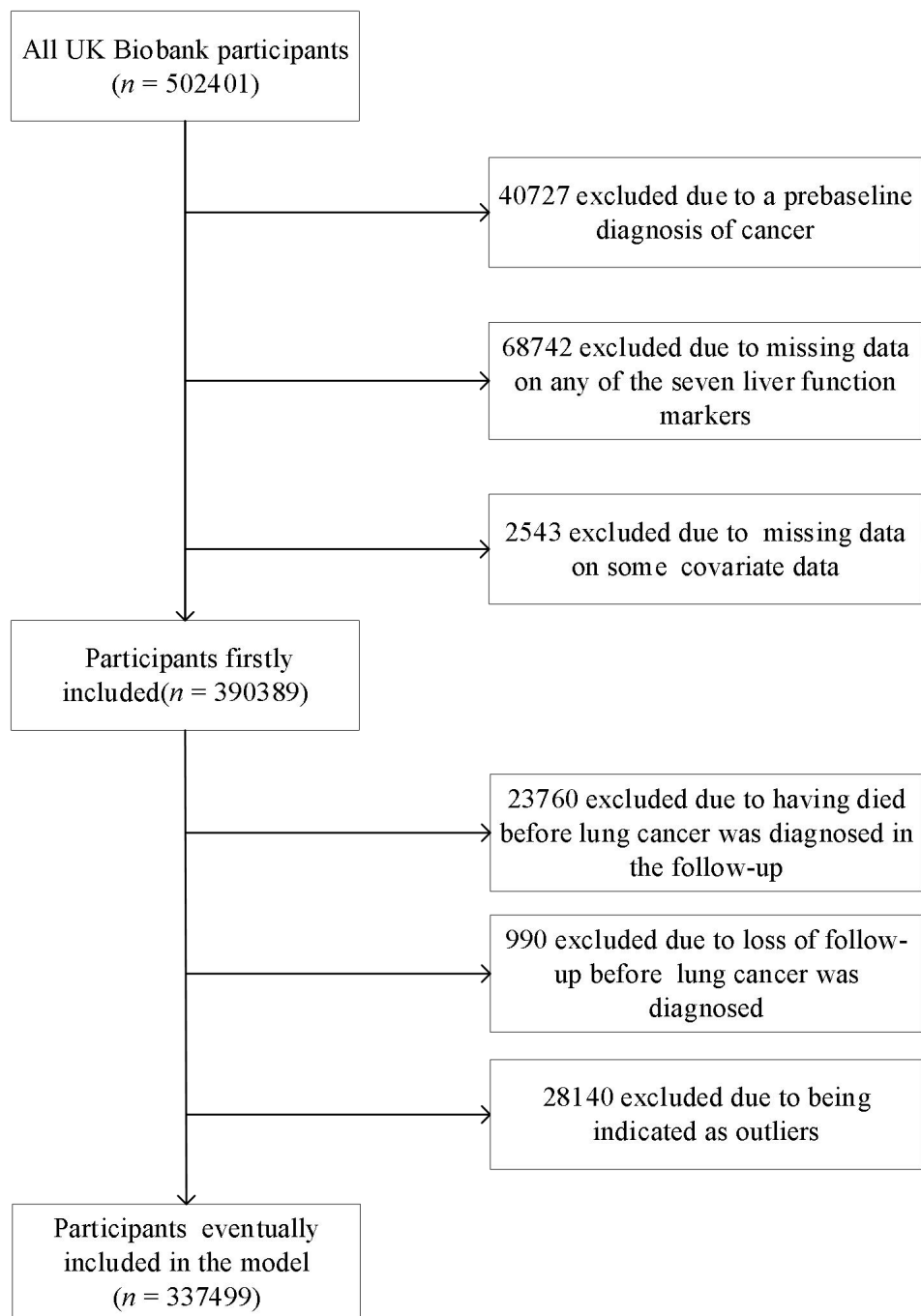

Supplement: Supplementary file 1 — Figure S1 Flowchart of participants’ inclusion in the UK Biobank. [file CRJ-18-e70042-s005.pdf]

Supplementary Figure 2

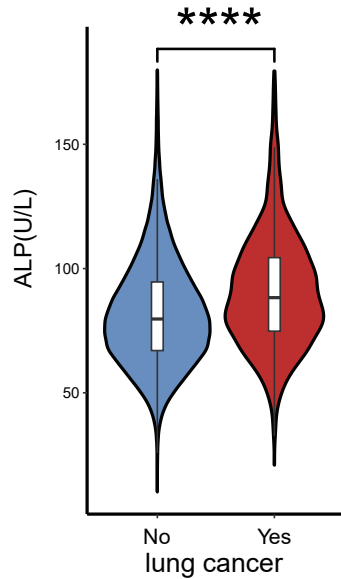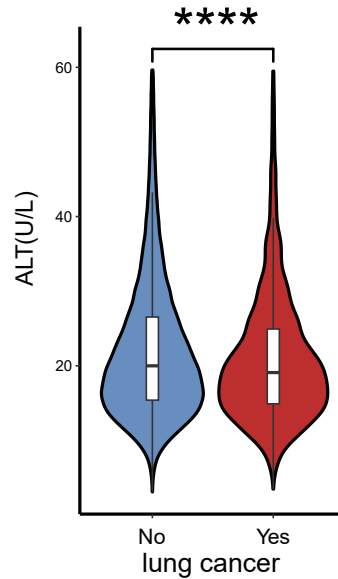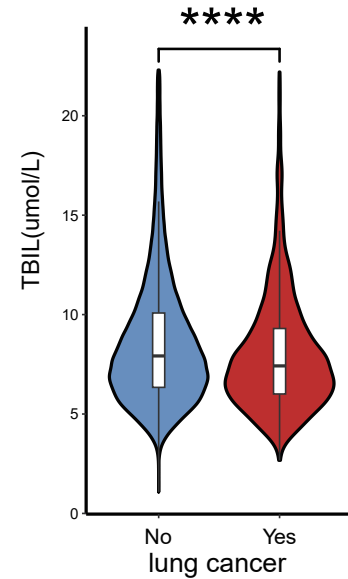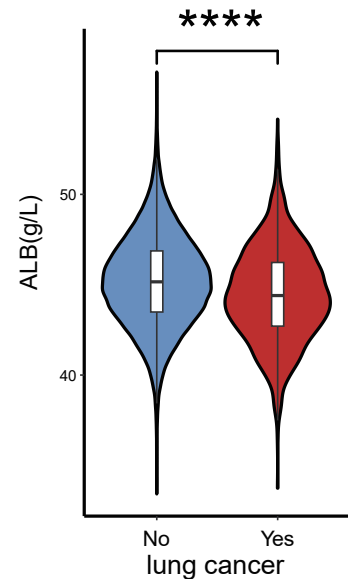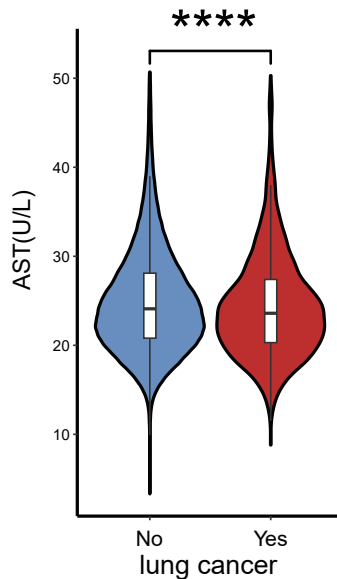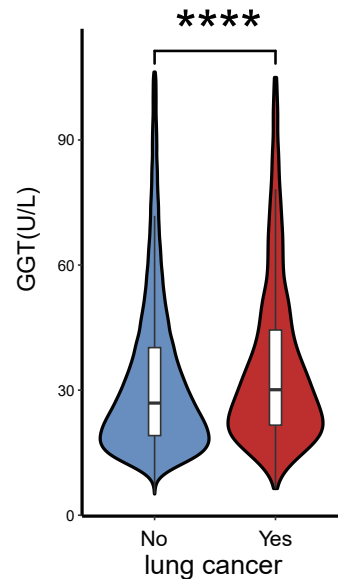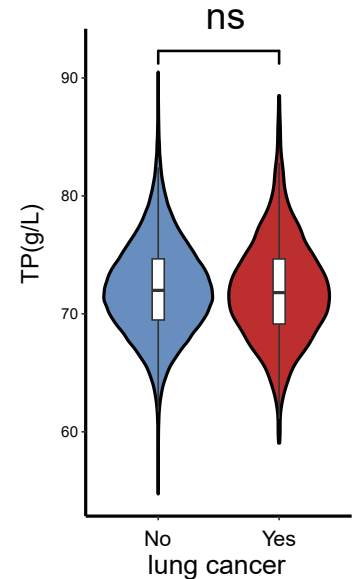

Supplement: Supplementary file 2 — Figure S2 The liver enzymes of smokers with lung cancer compared with the liver enzymes of smokers without lung cancer. [file CRJ-18-e70042-s002.pdf]

Supplementary Figure 3

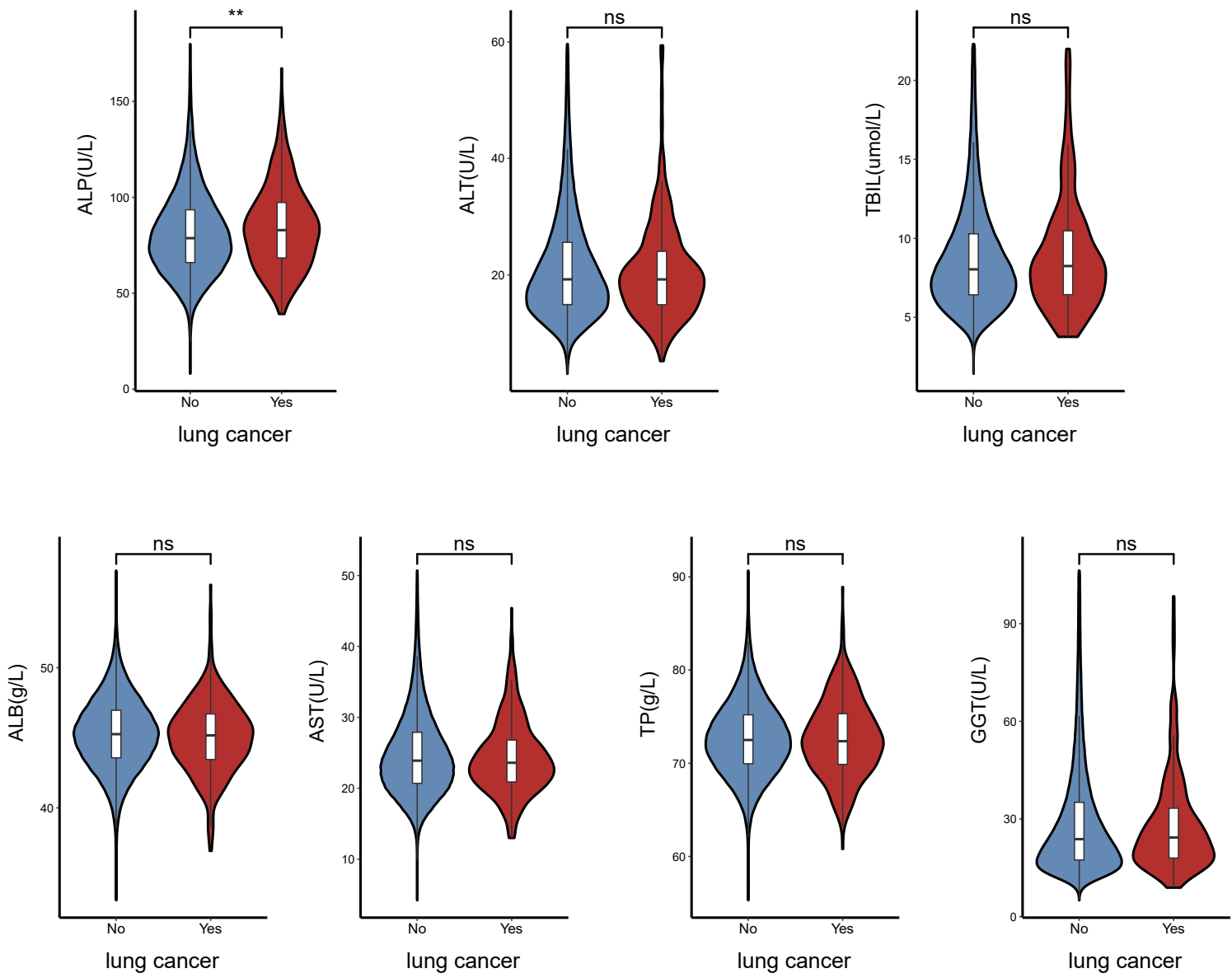

Supplement: Supplementary file 3 — Figure S3 The liver enzymes of nonsmokers with lung cancer compared with the liver enzymes of nonsmokers without lung cancer. [file CRJ-18-e70042-s004.pdf]
