# Supplementary material for: Liver Function Biomarkers and Lung Cancer Risk: A Prospective Cohort Study in the UK Biobank
Source: Clin Respir J. 2024 Dec 25;18(12):e70042. doi: 10.1111/crj.70042 (PMC11669495; doi:10.1111/crj.70042)
Supplement: Supplementary file 4 — Figure S4 The appearance of the web‐based personalized lung cancer risk prediction model. [file CRJ-18-e70042-s001.pdf]

Supplementary Figure 4

Nomogram for preoperative estimation of lung cancer risk

This model consists of seven liver function biomarkers (ALP, ALT, TBIL, ALB, AST, GGT and TP)and seven traditional predictors (age, sex, history of hay fever and/or allergic rhinitis and/or eczma, history of emphyse and/or machronic and/or bronchitis, family history of lung cancer, FEV1 and smoking-packyears). It discriminated well between incident lung cancer cases and non-cases, highlighting its clinical predictive value for early detection in high-risk population. It is the first to assess the predictive ability of liver function biomarkers in lung cancer risk model.

You have 1.1% possibility of lung cancer

See details below.

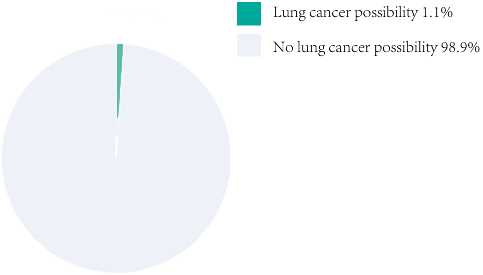

**Age(years)**	39–72 (slider)	50 years			
**Sex**	Male	Female			
**FEV1(liters)** Forced expiratory volume	<3	3~4	>4	Unkown	
**History of hay fever and/or allergic rhinitis and/or eczma**	No	Yes			
**Family history of lung cancer**	No	Yes			
**History of emphyse and/or machronic and/or bronchitis**	No	Yes			
**Smoking-packyears**	<10	10~20	>20		
**ALB(g/L)** Albumin(g/L)	<40	40~43	43~46	46~49	>49
**ALP(U/L)** Alkaline phosphatase(U/L)	<55	55~75	75~95	95~115	>115
**ALT(U/L)** Alanine transaminase (U/L)	<10	10~20	20~30	30~40	>40
**AST(U/L)** Aspartate transaminase(U/L)	<15	15~25	25~35	35~45	>45
**GGT(U/L)** Gamma glutamyltransfeiase (U/L)	<15	15~25	25~35	35~45	>45
**TBIL (μmol/L)** Total bilirubin (μmol/L)	<5	5~7.5	7.5~10	10~12	>12.5
**TP(g/L)** Total protein(g/L)	<62	62~68	68~74	74~80	>80
