# Supplementary material for: Liver Function Biomarkers and Lung Cancer Risk: A Prospective Cohort Study in the UK Biobank
Source: Clin Respir J. 2024 Dec 25;18(12):e70042. doi: 10.1111/crj.70042 (PMC11669495; doi:10.1111/crj.70042)
Supplement: Supplementary file 5 — Table S1 The average within‐laboratory coefficient of variation (CV) in quality‐control samples. Table S2. Normal range for levels of liver function biomarkers and the percentage of samples within the normal range. Table S3. Details for the GWAS information of exposure in MR analysis. Table S4. Details for the GWAS information of outcome in MR analysis. Table S5. Intraclass correlation coefficients of circulating liver function markers measured in repeated samples. Table S6. The hazard ratios (HRs) with 95% confidence intervals (95% CI) between various liver function biomarkers and lung cancer in sensitivity analyses. Table S7. The hazard risk (HR) with 95% confidence intervals (95% CI) between various liver function biomarkers and lung cancer by stratifying groups into smoker and nonsmoker using the full adjusted Cox proportional hazards model. Table S8. The hazard ratios (HRs) with 95% confidence intervals (95% CIs) between various liver function biomarkers and lung cancer stratifying by age (less than 60 years old vs. 60 years old or older) using the full adjusted Cox proportional hazards model. Table S9. The hazard ratios (HRs) with 95% confidence intervals (95% CIs) between various liver function biomarkers and lung cancer stratifying by sex using the full adjusted Cox proportional hazards model. Table S10. 93 SNPs associated with smoking initiation. Table S11. Detailed information on the SNPs instruments for the MR analysis of smoking initiation and alkaline phosphatase (ALP). Table S12. Detailed information on the SNPs for the MR analysis of smoking initiation and alanine aminotransferase (ALT). Table S13. Detailed information on the SNPs for the MR analysis of smoking initiation and total bilirubin (TBIL). Table S14. Detailed information on the SNPs for the MR analysis of smoking initiation and albumin (ALB). Table S15. Detailed information on the SNPs for the MR analysis of smoking initiation and aspartate aminotransferase (AST). Table S16. Detailed informa [file CRJ-18-e70042-s003.docx]

**Supplementary Table S1.** The average within-laboratory coefficient of variation

(CV) in quality-control samples

| **Biomarker** | **The range of CV** |
| --- | --- |
| Alanine transaminase (ALT) | 1.2% - 2.9% |
| Aspartate transaminase (AST) | 1.3% - 2.1% |
| Total bilirubin (TBIL) | 1.5% - 1.9% |
| Gamma glutamyltransferase (GGT) | 1.4% - 2.8% |
| Alkaline phosphatase (ALP) | 2.8% - 3.1% |
| Total Protein (TP) | 1.1% - 1.2% |
| Albumin (ALB) | 2.1% - 2.2% |

**Supplementary Table S2.** Normal range for levels of liver function biomarkers and the percentage of samples within the normal range

| **Biomarker** | **Normal range** | **Percentage of samples within the normal range** |
| --- | --- | --- |
| Alanine transaminase (ALT) | 7-52 U/L | 98.5% |
| Aspartate transaminase (AST) | 13-39 U/L | 97.10% |
| Total bilirubin (TBIL) | 5-17 μmol/L | 90.00% |
| Gamma glutamyltransferase (GGT) | 9-64 U/L | 93.10% |
| Alkaline phosphatase (ALP) | 34-104 U/L | 100% |
| Total Protein (TP) | 64-89 g/L | 98.80% |
| Albumin (ALB) | 35-57 g/L | 99.40% |

**Supplementary Table S3.** Details for the GWAS information of exposure in MR analysis

| **Exposure variables** | smoking initiation |
| --- | --- |
| **Source** | ieu-b-4877 |
| **Population** | European |
| **Sex** | Males and Females |
| **ncase** | 311629 |
| **ncontrol** | 321173 |
| **Sample size** | 607291 |
| **no.SNPs** | 11802365 |
| **Author** | Liu M |
| **Consortium** | GSCAN |
| **Ontology** | EFO:0005670 |
| **Build** | HG19/GRCh37 |

Sample size corresponds to the median effective sample size across SNPs, while ncases correspond to maximum reported ever smokers and ncontrols correspond to maximum reported never smokers in supplementary tables; the maximum reported sample size was 632802.

**Supplementary Table S4.** Details for the GWAS information of outcome in MR analysis

| **Variable** |  | **no.SNPs** | **Author** | **Source** |
| --- | --- | --- | --- | --- |
| Alkaline phosphatase(ALP) |  | 13586006 | Neale lab | ukb-d-30610_irnt |
| Alanine aminotransferase(ALT) |  | 13586000 | Neale lab | ukb-d-30620_irnt |
| Total bilirubin(TBIL) |  | 13585986 | Neale lab | ukb-d-30840_irnt |
| Albumin(ALB) |  | 13585334 | Neale lab | ukb-d-30600_irnt |
| Aspartate aminotransferase(AST) |  | 13586009 | Neale lab | ukb-d-30650_irnt |
| Gamma glutamyltransferase(GGT) |  | 13586026 | Neale lab | ukb-d-30730_irnt |
| Total protein(TP) |  | 13585298 | Neale lab | ukb-d-30860_irnt |

| Biomarker | Intraclass correlation coefficient(95% CI) |
| --- | --- |
| Alkaline phosphatase (ALP) | 0.84(0.83-0.84) |
| Alanine transaminase (ALT) | 0.66(0.65-0.67) |
| Total bilirubin (TBIL) | 0.85(0.84-0.86) |
| Albumin (ALB) | 0.62(0.60-0.63) |
| Gamma glutamyltransferase (GGT) | 0.78(0.77-0.78) |
| Aspartate transaminase (AST) | 0.65(0.64-0.67) |
| Total Protein (TP) | 0.62(0.61-0.64) |

**Supplementary Table S5.** Intraclass correlation coefficients of circulating liver function markers measured in repeated samples

**Supplementary Table S6.** The hazard ratios (HRs) with 95% confidence intervals (95%CI) between various liver function biomarkers and lung cancer in sensitivity analyses.

| **ALP** | **<55** | **55-75** | **75-95** | **95-115** | **>115** | **Per-SD increase** |
| --- | --- | --- | --- | --- | --- | --- |
| Excluding events occurred in the first two years of follow-up | 1(Ref) | 1.07(0.87-1.31) | 1.21(0.99-1.48) | 1.53(1.25-1.88) | 1.49(1.20-1.85) | 1.13(1.09-1.18) |
| Excluding liver disease | 1(Ref) | 1.06(0.88-1.29) | 1.24(1.03-1.50) | 1.57(1.29-1.90) | 1.56(1.27-1.91) | 1.15(1.11-1.19) |
| **ALT** | **<10** | **10-20** | **20-30** | **30-40** | **>40** | **Per-SD increase** |
| Excluding events occurred in the first two years of follow-up | 1(Ref) | 0.90(0.73-1.11) | 0.83(0.67-1.03) | 0.74(0.58-0.95) | 0.69(0.52-0.92) | 0.91(0.87-0.95) |
| Excluding liver disease | 1(Ref) | 0.91(0.74-1.10) | 0.83(0.67-1.01) | 0.74(0.59-0.93) | 0.68(0.51-0.89) | 0.90(0.86-0.94) |
| **TBIL** | **<5** | **5-7.5** | **7.5-10** | **10-12.5** | **>12.5** | **Per-SD increase** |
| Excluding events occurred in the first two years of follow-up | 1(Ref) | 0.92(0.81-1.06) | 0.85(0.73-0.98) | 0.86(0.73-1.02) | 0.72(0.60-0.87) | 0.92(0.88-0.86) |
| Excluding liver disease | 1(Ref) | 0.88(0.78-1.00) | 0.83(0.72-0.95) | 0.82(0.70-0.96) | 0.72(0.60-0.85) | 0.92(0.88-0.86) |
| **ALB(g/L)** | **<40** | **40-43** | **43-46** | **46-49** | **>49** |  |
| Excluding events occurred in the first two years of follow-up | 1(Ref) | 0.92(0.74-1.15) | 0.78(0.63-0.97) | 0.71(0.57-0.88) | 0.62(0.47-0.82) |  |
| Excluding liver disease | 1(Ref) | 0.85(0.70-1.04) | 0.70(0.58-0.86) | 0.64(0.53-0.79) | 0.55(0.42-0.71) |  |
| **GGT(U/L)** | **<15** | **15-25** | **25-35** | **35-45** | **>45** |  |
| Excluding events occurred in the first two years of follow-up | 0.81(0.68-0.97) | 0.90(0.82-1.00) | 1(Ref) | 0.97(0.85-1.10) | 1.04(0.93-1.17) |  |
| Excluding liver disease | 0.80(0.68-0.95) | 0.90(0.82-0.99) | 1(Ref) | 0.98(0.87-1.10) | 1.04(0.94-1.16) |  |
| **AST(U/L)** | **<15** | **15-25** | **25-35** | **35-45** | **>45** |  |
| Excluding events occurred in the first two years of follow-up | 1(Ref) | 0.74(0.55-1.00) | 0.66(0.48-0.88) | 0.57(0.40-0.81) | 0.78(0.45-1.33) |  |
| Excluding liver disease | 1(Ref) | 0.68(0.52-0.90) | 0.61(0.46-0.80) | 0.52(0.37-0.71) | 0.71(0.43-1.18) |  |
| **TP(g/L)** | **<62** | **62-68** | **68-74** | **74-80** | **>80** |  |
| Excluding events occurred in the first two years of follow-up | 1.20(0.66-2.17) | 1.06(0.95-1.18) | 1(Ref) | 1.07(0.98-1.17) | 1.27(1.04-1.55) |  |
| Excluding liver disease | 1.46(0.88-2.43) | 1.03(0.93-1.15) | 1(Ref) | 1.06(0.97-1.15) | 1.31(1.08-1.58) |  |

**Supplementary Table 7.** The hazard risk (HR) with 95% confidence intervals (95%CI) between various liver function biomarkers and lung cancer by stratifying groups into smoker and non-smoker using the full adjusted Cox proportional hazard model.

|  |  |  |
| --- | --- | --- |
|  | **smoker** | **nonsmoker** |
|  | **HR(95%CI)** | **HR(95%CI)** |
| **ALP(U/L)** | ***P* of interaction = 0.003** |  |
| <55 | 1(Ref) | 1(Ref) |
| 55-75 | 1.20(0.96-1.49) | 0.82(0.55-1.22) |
| 75-95 | 1.53(1.23-1.90) | 0.88(0.59-1.29) |
| 95-115 | 2.12(1.70-2.64) | 0.89(0.58-1.35) |
| >115 | 2.14(1.69-2.69) | 0.98(0.61-1.57) |
| **ALT(U/L)** | ***P* of interaction = 0.377** |  |
| <10 | 1(Ref) | 1(Ref) |
| 10-20 | 0.86(0.69-1.06) | 0.72(0.45-1.15) |
| 20-30 | 0.70(0.56-0.87) | 0.75(0.46-1.21) |
| 30-40 | 0.62(0.48-0.79) | 0.63(0.36-1.11) |
| >40 | 0.58(0.43-0.77) | 0.37(0.17-0.81) |
| **TBIL(μmol/L)** | ***P* of interaction = 0.006** |  |
| <5 | 1(Ref) | 1(Ref) |
| 5-7.5 | 0.84(0.73-0.97) | 0.6(0.43-0.85) |
| 7.5-10 | 0.69(0.6-0.8) | 0.69(0.48-0.97) |
| 10-12.5 | 0.66(0.55-0.78) | 0.73(0.49-1.09) |
| >12.5 | 0.51(0.42-0.62) | 0.81(0.54-1.22) |
| **ALB(g/L)** | ***P* of interaction = 0.014** |  |
| <40 | 1(Ref) | 1(Ref) |
| 40-43 | 0.84(0.68-1.04) | 0.77(0.42-1.42) |
| 43-46 | 0.66(0.53-0.81) | 0.87(0.48-1.56) |
| 46-49 | 0.56(0.45-0.70) | 0.86(0.48-1.57) |
| >49 | 0.48(0.37-0.64) | 0.67(0.33-1.38) |
| **GGT(U/L)** | ***P* of interaction = 0.010** |  |
| <15 | 0.71(0.59-0.85) | 0.80(0.56-1.14) |
| 15-25 | 0.86(0.78-0.96) | 0.94(0.74-1.20) |
| 25-35 | 1(Ref) | 1(Ref) |
| 35-45 | 0.98(0.86-1.11) | 0.93(0.67-1.30) |
| >45 | 1.08(0.97-1.21) | 0.71(0.51-0.98) |
| **AST(U/L)** | ***P* of interaction = 0.138** |  |
| <15 | 1(Ref) | 1(Ref) |
| 15-25 | 0.63(0.46-0.85) | 0.40(0.21-0.76) |
| 25-35 | 0.5(0.37-0.68) | 0.33(0.17-0.63) |
| 35-45 | 0.42(0.29-0.59) | 0.27(0.12-0.58) |
| >45 | 0.6(0.35-1.02) | 0.12(0.02-0.97) |
| **TP(g/L)** | ***P* of interaction = 0.907** |  |
| <62 | 1.56(0.92-2.64) | 0.84(0.12-6.00) |
| 62-68 | 1.13(1.01-1.26) | 1.01(0.75-1.37) |
| 68-74 | 1(Ref) | 1(Ref) |
| 74-80 | 0.99(0.91-1.09) | 1.02(0.82-1.26) |
| >80 | 1.22(0.99-1.50) | 1.15(0.73-1.82) |

**Supplementary Table 8.** The hazard ratios (HRs) with 95% confidence intervals (95%CIs) between various liver function biomarkers and lung cancer stratifying by age (less than 60 years old vs. 60 years old or older) using the full adjusted Cox proportional hazard model.

|  |  | **Age at baseline** | **Age at baseline** |
| --- | --- | --- | --- |
|  |  | **<60** | **>=60** |
| **ALP(U/L)** |  | ***P* of interaction = 0.622** |  |
| <55 |  | 1(Ref) | 1(Ref) |
| 55-75 |  | 1.07(0.78-1.48) | 1.15(0.90-1.46) |
| 75-95 |  | 1.50(1.10-2.05) | 1.31(1.04-1.66) |
| 95-115 |  | 2.15(1.56-2.96) | 1.62(1.27-2.07) |
| >115 |  | 2.15(1.51-3.05) | 1.60(1.24-2.07) |
| **ALT(U/L)** |  | ***P* of interaction = 0.163** |  |
| <10 |  | 1(Ref) | 1(Ref) |
| 10-20 |  | 1.07(0.77-1.49) | 0.87(0.68-1.10) |
| 20-30 |  | 0.91(0.64-1.29) | 0.81(0.63-1.04) |
| 30-40 |  | 0.72(0.48-1.09) | 0.74(0.56-0.97) |
| >40 |  | 0.81(0.51-1.26) | 0.58(0.41-0.82) |
| **TBIL(μmol/L)** |  | ***P* of interaction = 0.437** |  |
| <5 |  | 1(Ref) | 1(Ref) |
| 5-7.5 |  | 0.85(0.68-1.05) | 0.94(0.80-1.10) |
| 7.5-10 |  | 0.75(0.59-0.95) | 0.91(0.77-1.08) |
| 10-12.5 |  | 0.79(0.59-1.04) | 0.89(0.74-1.08) |
| >12.5 |  | 0.74(0.54-1.01) | 0.75(0.61-0.92) |
| **ALB(g/L)** |  | ***P* of interaction = 0.262** |  |
| <40 |  | 1(Ref) | 1(Ref) |
| 40-43 |  | 0.67(0.46-0.99) | 0.92(0.73-1.16) |
| 43-46 |  | 0.57(0.39-0.83) | 0.74(0.59-0.93) |
| 46-49 |  | 0.45(0.31-0.66) | 0.70(0.55-0.88) |
| >49 |  | 0.34(0.21-0.55) | 0.62(0.45-0.84) |
| **GGT(U/L)** |  | ***P* of interaction =0.012** |  |
| <15 |  | 0.89(0.68-1.15) | 0.68(0.55-0.84) |
| 15-25 |  | 0.97(0.80-1.17) | 0.86(0.77-0.96) |
| 25-35 |  | 1(Ref) | 1(Ref) |
| 35-45 |  | 1.16(0.92-1.46) | 0.9(0.78-1.03) |
| >45 |  | 0.98(0.80-1.22) | 1.05(0.93-1.19) |
| **AST(U/L)** |  | ***P* of interaction = 0.075** |  |
| <15 |  | 1(Ref) | 1(Ref) |
| 15-25 |  | 0.60(0.42-0.87) | 0.93(0.61-1.40) |
| 25-35 |  | 0.54(0.37-0.79) | 0.84(0.56-1.28) |
| 35-45 |  | 0.42(0.25-0.69) | 0.74(0.47-1.16) |
| >45 |  | 0.67(0.28-1.62) | 0.9(0.47-1.73) |
| **TP(g/L)** |  | ***P* of interaction < 0.001** |  |
| <62 |  | 3.69(1.91-7.15) | 0.74(0.33-1.65) |
| 62-68 |  | 1.19(0.99-1.44) | 1.00(0.89-1.14) |
| 68-74 |  | 1(Ref) | 1(Ref) |
| 74-80 |  | 0.78(0.66-1.93) | 1.16(1.05-1.28) |
| >80 |  | 1.13(0.80-1.59) | 1.32(1.06-1.65) |

**Supplementary Table 9.** The hazard ratios (HRs) with 95% confidence intervals (95%CIs) between various liver function biomarkers and lung cancer stratifying by sex using the full adjusted Cox proportional hazard model.

|  | **Female** | **male** |
| --- | --- | --- |
|  |  |  |
| **ALP(U/L)** | ***P* of interaction = 0.073** |  |
| <55 | 1(Ref) | 1(Ref) |
| 55-75 | 0.97(0.73-1.27) | 1.17(0.89-1.53) |
| 75-95 | 1.15(0.88-1.50) | 1.35(1.03-1.76) |
| 95-115 | 1.29(0.98-1.69) | 1.92(1.46-1.52) |
| >115 | 1.30(0.97-1.73) | 1.83(1.36-2.46) |
| **ALT(U/L)** | ***P* of interaction = 0.134** |  |
| <10 | 1(Ref) | 1(Ref) |
| 10-20 | 0.97(0.76-0.24) | 0.74(0.54-1.02) |
| 20-30 | 0.93(0.71-1.20) | 0.65(0.47-0.89) |
| 30-40 | 0.81(0.59-0.12) | 0.60(0.42-0.85) |
| >40 | 0.98(0.66-1.47) | 0.48(0.32-0.71) |
| **TBIL(μmol/L)** | ***P* of interaction = 0.818** |  |
| <5 | 1(Ref) | 1(Ref) |
| 5-7.5 | 0.91(0.78-1.06) | 0.82(0.65-1.04) |
| 7.5-10 | 0.86(0.72-1.02) | 0.78(0.61-0.98) |
| 10-12.5 | 0.93(0.75-1.17) | 0.73(0.57-0.95) |
| >12.5 | 0.78(0.60-1.02) | 0.64(0.49-0.84) |
| **ALB(g/L)** | ***P* of interaction = 0.687** |  |
| <40 | 1(Ref) | 1(Ref) |
| 40-43 | 0.91(0.68-1.21) | 0.81(0.61-1.07) |
| 43-46 | 0.79(0.60-1.05) | 0.64(0.49-0.84) |
| 46-49 | 0.73(0.55-0.98) | 0.59(0.44-0.78) |
| >49 | 0.56(0.39-0.83) | 0.54(0.38-0.77) |
| **GGT(U/L)** | ***P* of interaction = 0.353** |  |
| <15 | 0.76(0.63-0.92) | 0.88(0.60-1.28) |
| 15-25 | 0.82(0.72-0.94) | 0.99(0.86-1.14) |
| 25-35 | 1(Ref) | 1(Ref) |
| 35-45 | 0.94(0.77-0.13) | 1.00(0.86-1.17) |
| >45 | 0.97(0.82-1.15) | 1.10(0.97-1.26) |
| **AST(U/L)** | ***P* of interaction = 0.427** |  |
| <15 | 1(Ref) | 1(Ref) |
| 15-25 | 0.75(0.52-1.07) | 0.58(0.38-0.89) |
| 25-35 | 0.65(0.45-0.95) | 0.53(0.34-0.81) |
| 35-45 | 0.61(0.39-0.97) | 0.43(0.27-0.69) |
| >45 | 1.15(0.58-2.30) | 0.44(0.21-0.93) |
| **TP(g/L)** | ***P* of interaction = 0.323** |  |
| <62 | 1.51(0.75-3.02) | 1.40(0.66-2.94) |
| 62-68 | 0.97(0.84-1.13) | 1.11(0.96-1.28) |
| 68-74 | 1(Ref) | 1(Ref) |
| 74-80 | 0.94(0.83-1.07) | 1.17(1.04-1.31) |
| >80 | 1.20(0.91-1.58) | 1.40(1.09-1.80) |
|  |  |  |

**Supplementary Table 10.** 93 SNPs associated with smoking initiation

| **se** | **pval** | **samplesize** | **SNP** | **effect allele** | **other allele** | **eaf.exposure** | **R2** | **F** |
| --- | --- | --- | --- | --- | --- | --- | --- | --- |
| 0.0038983 | 8.12E-18 | 632802 | rs3001723 | A | G | 0.321 | 0.000489554 | 309.9412972 |
| 0.00355604 | 1.14E-11 | 632802 | rs7555507 | T | C | 0.496 | 0.000291457 | 184.4879949 |
| 0.0043955 | 3.36E-09 | 632802 | rs6669839 | T | C | 0.204 | 0.000219612 | 139.001262 |
| 0.0035682 | 4.22E-10 | 632802 | rs12042107 | C | T | 0.527 | 0.000247551 | 156.6890537 |
| 0.00358602 | 3.61E-13 | 632802 | rs2186122 | T | A | 0.561 | 0.000334438 | 211.7034536 |
| 0.00361329 | 2.80E-09 | 632802 | rs301805 | G | T | 0.559 | 0.000227227 | 143.8217886 |
| 0.00533915 | 6.52E-10 | 632802 | rs12025237 | C | A | 0.124 | 0.000236572 | 149.7379331 |
| 0.00370828 | 3.00E-08 | 632802 | rs2050586 | C | G | 0.355 | 0.000193331 | 122.363718 |
| 0.00447842 | 3.03E-08 | 632802 | rs2046850 | T | C | 0.187 | 0.00018722 | 118.4949903 |
| 0.00473279 | 6.73E-14 | 632802 | rs6728726 | C | T | 0.829 | 0.00035627 | 225.5277756 |
| 0.00365894 | 2.03E-08 | 632802 | rs78411160 | C | A | 0.631 | 0.000196383 | 124.2958162 |
| 0.00405809 | 3.16E-08 | 632802 | rs6433897 | C | T | 0.754 | 0.00018694 | 118.318057 |
| 0.00373855 | 3.36E-16 | 632802 | rs266047 | A | G | 0.529 | 0.000463858 | 293.6657274 |
| 0.00443619 | 1.32E-08 | 632802 | rs4674993 | G | A | 0.207 | 0.000208687 | 132.0845823 |
| 0.00359627 | 1.50E-15 | 632802 | rs578584 | T | A | 0.605 | 0.000393137 | 248.8748606 |
| 0.00423093 | 2.43E-09 | 632802 | rs35702515 | T | G | 0.162 | 0.000173026 | 109.5100242 |
| 0.0035563 | 3.56E-24 | 632802 | rs13030994 | A | G | 0.485 | 0.000650748 | 412.0615315 |
| 0.00358234 | 1.25E-14 | 632802 | rs12474587 | T | G | 0.404 | 0.000367714 | 232.7752209 |
| 0.00492533 | 3.27E-08 | 632802 | rs2107300 | G | C | 0.845 | 0.000193815 | 122.6701215 |
| 0.0037281 | 1.88E-09 | 632802 | rs7585579 | G | C | 0.505 | 0.000250846 | 158.7751471 |
| 0.00356484 | 1.68E-11 | 632802 | rs1445649 | C | T | 0.525 | 0.000287117 | 181.7399645 |
| 0.00368905 | 1.91E-17 | 632802 | rs6788098 | T | A | 0.623 | 0.000461558 | 292.2088674 |
| 0.00375292 | 4.78E-10 | 632802 | rs12632110 | G | A | 0.647 | 0.00024962 | 157.9988758 |
| 0.00457843 | 3.51E-09 | 632802 | rs11712680 | C | A | 0.174 | 0.000210289 | 133.0989315 |
| 0.00491232 | 3.12E-11 | 632802 | rs1154693 | G | A | 0.856 | 0.000262349 | 166.0580193 |
| 0.00365268 | 2.83E-08 | 632802 | rs66680800 | T | G | 0.397 | 0.00019675 | 124.5276513 |
| 0.00356289 | 2.97E-08 | 632802 | rs1869243 | C | T | 0.481 | 0.000194574 | 123.1504797 |
| 0.00414237 | 6.32E-09 | 632802 | rs9835772 | T | A | 0.235 | 0.000207916 | 131.5967309 |
| 0.00403803 | 4.37E-09 | 632802 | rs962625 | G | A | 0.24 | 0.000205218 | 129.8883577 |
| 0.00429163 | 1.53E-09 | 632802 | rs993700 | C | T | 0.766 | 0.000240998 | 152.5400224 |
| 0.00366263 | 2.14E-10 | 632802 | rs13145728 | C | G | 0.358 | 0.000248507 | 157.294379 |
| 0.00364155 | 6.65E-12 | 632802 | rs10001365 | A | G | 0.405 | 0.000301021 | 190.5435622 |
| 0.00358893 | 7.20E-09 | 632802 | rs1160685 | G | C | 0.478 | 0.000215329 | 136.2892952 |
| 0.00407356 | 3.25E-09 | 632802 | rs6893752 | G | A | 0.766 | 0.000208205 | 131.7794192 |
| 0.00502051 | 3.42E-11 | 632802 | rs12186738 | T | G | 0.154 | 0.000288324 | 182.5039104 |
| 0.00415673 | 3.00E-09 | 632802 | rs1385108 | T | C | 0.239 | 0.000221237 | 140.0300157 |
| 0.00371058 | 6.08E-14 | 632802 | rs4044321 | G | A | 0.642 | 0.000356319 | 225.5593296 |
| 0.00356881 | 1.22E-14 | 632802 | rs4352629 | T | C | 0.492 | 0.000378809 | 239.8014294 |
| 0.00528628 | 5.02E-10 | 632802 | rs72789632 | T | C | 0.12 | 0.000228405 | 144.5676531 |
| 0.003986 | 3.47E-12 | 632802 | rs9401770 | A | G | 0.273 | 0.000305245 | 193.2180859 |
| 0.00442796 | 1.08E-08 | 632802 | rs222449 | T | A | 0.793 | 0.000210489 | 133.2251655 |
| 0.00405809 | 1.93E-08 | 632802 | rs3800227 | G | A | 0.701 | 0.000218147 | 138.0736979 |
| 0.00355561 | 6.62E-09 | 632802 | rs10498846 | T | C | 0.473 | 0.000211773 | 134.0382751 |
| 0.00483712 | 2.16E-17 | 632802 | rs240963 | C | T | 0.836 | 0.000461943 | 292.452326 |
| 0.00480127 | 1.44E-09 | 632802 | rs12333760 | C | T | 0.204 | 0.00027401 | 173.441219 |
| 0.00355741 | 2.75E-14 | 632802 | rs10233018 | G | A | 0.503 | 0.000366352 | 231.9126284 |
| 0.00366263 | 5.00E-09 | 632802 | rs10279261 | A | G | 0.619 | 0.000216401 | 136.9684955 |
| 0.00360938 | 1.75E-08 | 632802 | rs10260968 | A | G | 0.597 | 0.000198716 | 125.7727333 |
| 0.00404299 | 1.34E-09 | 632802 | rs12112638 | G | A | 0.275 | 0.000239858 | 151.8185349 |
| 0.00355661 | 3.35E-12 | 632802 | rs4236259 | G | T | 0.499 | 0.000306748 | 194.1696808 |
| 0.00373402 | 4.70E-10 | 632802 | rs2140114 | T | C | 0.518 | 0.000270142 | 170.9922445 |
| 0.00373982 | 3.74E-09 | 632802 | rs3801289 | C | A | 0.351 | 0.00022175 | 140.3545452 |
| 0.0044613 | 3.42E-17 | 632802 | rs1565735 | A | T | 0.212 | 0.000472807 | 299.3335075 |
| 0.00388691 | 1.04E-11 | 632802 | rs1899896 | T | C | 0.286 | 0.000285682 | 180.8313629 |
| 0.00355604 | 3.90E-14 | 632802 | rs13261666 | T | G | 0.522 | 0.00036096 | 228.4977015 |
| 0.00363668 | 2.43E-08 | 632802 | rs12545053 | G | A | 0.397 | 0.000196928 | 124.6407297 |
| 0.00402823 | 1.18E-08 | 632802 | rs2631024 | G | A | 0.737 | 0.000204441 | 129.3964874 |
| 0.00356244 | 7.46E-10 | 632802 | rs4543592 | C | T | 0.468 | 0.000239508 | 151.59703 |
| 0.00356645 | 4.16E-09 | 632802 | rs2378662 | A | G | 0.556 | 0.000216661 | 137.1329302 |
| 0.00453171 | 1.81E-08 | 632802 | rs10114490 | A | G | 0.198 | 0.000206754 | 130.8609784 |
| 0.00414505 | 7.35E-09 | 632802 | rs10905461 | C | T | 0.718 | 0.000232386 | 147.0881916 |
| 0.00355815 | 8.26E-13 | 632802 | rs7921378 | C | G | 0.463 | 0.000322334 | 204.0384254 |
| 0.0050491 | 6.27E-15 | 632802 | rs12356821 | C | G | 0.14 | 0.000373239 | 236.2739864 |
| 0.00372727 | 1.84E-12 | 632802 | rs10159545 | G | C | 0.375 | 0.000323001 | 204.4607623 |
| 0.00370828 | 3.21E-08 | 632802 | rs9423279 | G | C | 0.641 | 0.000193664 | 122.5744317 |
| 0.00363668 | 2.71E-33 | 632802 | rs7938812 | G | T | 0.424 | 0.00093669 | 593.293316 |
| 0.00457843 | 3.77E-12 | 632802 | rs6265 | T | C | 0.203 | 0.000326937 | 206.9535076 |
| 0.00428466 | 1.56E-08 | 632802 | rs7929518 | G | A | 0.765 | 0.000211223 | 133.6904293 |
| 0.00364321 | 1.55E-08 | 632802 | rs4523689 | G | A | 0.408 | 0.000205178 | 129.8630222 |
| 0.00357892 | 4.85E-09 | 632802 | rs11057005 | G | A | 0.43 | 0.000214735 | 135.9136897 |
| 0.00393413 | 3.58E-08 | 632802 | rs4759228 | C | G | 0.27 | 0.000185476 | 117.3910023 |
| 0.00395946 | 7.31E-10 | 632802 | rs7969559 | G | A | 0.688 | 0.000255084 | 161.4585037 |
| 0.00492533 | 7.06E-09 | 632802 | rs1971318 | T | C | 0.141 | 0.00019686 | 124.5975393 |
| 0.00433474 | 3.58E-09 | 632802 | rs7322872 | T | C | 0.782 | 0.000222946 | 141.1114201 |
| 0.0035765 | 3.23E-09 | 632802 | rs3904512 | A | G | 0.429 | 0.000219336 | 138.8261582 |
| 0.00355789 | 3.82E-08 | 632802 | rs9540729 | T | A | 0.501 | 0.000191143 | 120.9787297 |
| 0.00454745 | 3.99E-08 | 632802 | rs76214862 | C | A | 0.202 | 0.000201339 | 127.4327677 |
| 0.00452262 | 1.06E-10 | 632802 | rs12441907 | A | C | 0.186 | 0.000258276 | 163.4795347 |
| 0.00359095 | 2.64E-16 | 632802 | rs1435741 | A | G | 0.425 | 0.00042289 | 267.7180154 |
| 0.00365894 | 2.26E-08 | 632802 | rs4785836 | C | T | 0.398 | 0.000200799 | 127.0913235 |
| 0.0041686 | 2.77E-09 | 632802 | rs7197072 | T | C | 0.238 | 0.000222493 | 140.8247066 |
| 0.00358893 | 1.67E-09 | 632802 | rs1050847 | T | C | 0.505 | 0.000233756 | 147.9552866 |
| 0.00436473 | 4.54E-08 | 632802 | rs4781977 | C | T | 0.205 | 0.000185669 | 117.5131549 |
| 0.00360561 | 2.23E-08 | 632802 | rs11078713 | G | A | 0.454 | 0.000201735 | 127.6835085 |
| 0.00365532 | 1.43E-08 | 632802 | rs7224742 | T | C | 0.595 | 0.000206708 | 130.8320783 |
| 0.00361066 | 2.43E-08 | 632802 | rs11658881 | G | A | 0.418 | 0.000197271 | 124.8575701 |
| 0.00358602 | 7.97E-09 | 632802 | rs6508144 | G | C | 0.563 | 0.000210711 | 133.3661828 |
| 0.00409477 | 1.43E-09 | 632802 | rs11872397 | A | G | 0.252 | 0.000231351 | 146.4329343 |
| 0.00483712 | 2.75E-08 | 632802 | rs72896886 | C | G | 0.144 | 0.000178238 | 112.8088802 |
| 0.00825958 | 1.94E-09 | 632802 | rs76608582 | A | C | 0.0389 | 0.00018364 | 116.2286478 |
| 0.0038234 | 3.65E-09 | 632802 | rs1555445 | T | A | 0.337 | 0.000227327 | 143.8853556 |
| 0.00526909 | 2.76E-08 | 632802 | rs117143374 | C | T | 0.12 | 0.000181186 | 114.6750469 |
| 0.00366078 | 4.85E-08 | 632802 | rs134529 | C | T | 0.349 | 0.000181469 | 114.8541102 |

**Supplementary Table 11.** Detailed information on the SNPs instruments for the MR analysis of smoking initiation and Alkaline phosphatase(ALP).

Removing the following SNPs for being palindromic with intermediate allele frequencies:

rs1160685, rs2186122, rs578584, rs6508144, rs7585579, rs7921378, rs9540729

| **SNP** | **beta.outcome** | **eaf.outcome** | **chr** | **pos** | **se.outcome** | **samplesize.**  **outcome** | **pval.outcome** |
| --- | --- | --- | --- | --- | --- | --- | --- |
| rs10001365 | -0.00079483 | 0.38617 | 4 | 147797214 | 0.0023907 | 344292 | 0.73953 |
| rs10114490 | -0.00059019 | 0.18552 | 9 | 11070165 | 0.0029931 | 344292 | 0.84368 |
| rs10159545 | 0.0023144 | 0.34793 | 10 | 21766969 | 0.0024617 | 344292 | 0.34713 |
| rs10233018 | 0.0023412 | 0.50413 | 7 | 117523709 | 0.0023304 | 344292 | 0.31507 |
| rs10260968 | -0.0029825 | 0.58882 | 7 | 1889773 | 0.0023628 | 344292 | 0.20685 |
| rs10279261 | 0.002507 | 0.61768 | 7 | 133589846 | 0.0024037 | 344292 | 0.29696 |
| rs10498846 | 0.0039893 | 0.50752 | 6 | 67405337 | 0.0023411 | 344292 | 0.0883731 |
| rs1050847 | -0.0039673 | 0.57911 | 16 | 87443734 | 0.002364 | 344292 | 0.0933104 |
| rs10905461 | -0.00086416 | 0.74427 | 10 | 8803551 | 0.0026701 | 344292 | 0.74621 |
| rs11057005 | -0.0023961 | 0.43786 | 12 | 16748721 | 0.0023602 | 344292 | 0.31001 |
| rs11078713 | 0.0071365 | 0.41763 | 17 | 7795972 | 0.0023628 | 344292 | 0.00252488 |
| rs1154693 | 0.00040717 | 0.85141 | 3 | 117804154 | 0.0032871 | 344292 | 0.90142 |
| rs1160685 | 0.0016118 | 0.44087 | 4 | 94052854 | 0.0023457 | 344292 | 0.49201 |
| rs11658881 | -0.010366 | 0.42026 | 17 | 2072949 | 0.0023609 | 344292 | 1.13E-05 |
| rs11712680 | 0.0030971 | 0.18623 | 3 | 75009019 | 0.0029897 | 344292 | 0.30024 |
| rs117143374 | 0.00034749 | 0.14188 | 21 | 40555561 | 0.003345 | 344292 | 0.91726 |
| rs11872397 | 0.0015586 | 0.25979 | 18 | 72535282 | 0.0026854 | 344292 | 0.56165 |
| rs12025237 | -0.0029412 | 0.12588 | 1 | 154205120 | 0.0035098 | 344292 | 0.40203 |
| rs12042107 | -0.0010162 | 0.54983 | 1 | 91196176 | 0.0023441 | 344292 | 0.664639 |
| rs12112638 | 0.0012285 | 0.26337 | 7 | 69735251 | 0.0026442 | 344292 | 0.642231 |
| rs12186738 | -0.0015472 | 0.14224 | 5 | 103816655 | 0.0033327 | 344292 | 0.64247 |
| rs12333760 | -0.0051012 | 0.16503 | 7 | 99185406 | 0.0031403 | 344292 | 0.10429 |
| rs12356821 | 0.0064017 | 0.14864 | 10 | 104563808 | 0.0033161 | 344292 | 0.0535513 |
| rs12441907 | 0.0020036 | 0.18911 | 15 | 83922387 | 0.0029979 | 344292 | 0.50393 |
| rs12474587 | -0.00035111 | 0.45073 | 2 | 162802993 | 0.0023432 | 344292 | 0.88089 |
| rs12545053 | -0.0092645 | 0.40326 | 8 | 65073605 | 0.0023782 | 344292 | 9.80E-05 |
| rs12632110 | -6.80E-05 | 0.65824 | 3 | 50224225 | 0.0024592 | 344292 | 0.97794 |
| rs13030994 | -0.00029178 | 0.48229 | 2 | 146143090 | 0.0023292 | 344292 | 0.90031 |
| rs13145728 | -0.0030911 | 0.38202 | 4 | 140927812 | 0.0023979 | 344292 | 0.19736 |
| rs13261666 | 3.78E-05 | 0.50399 | 8 | 59814666 | 0.002327 | 344292 | 0.98705 |
| rs134529 | -0.017846 | 0.38242 | 22 | 28781758 | 0.0023976 | 344292 | 9.85E-14 |
| rs1385108 | -0.0015019 | 0.24247 | 5 | 154839646 | 0.0027237 | 344292 | 0.58135 |
| rs1435741 | 0.0088003 | 0.42999 | 15 | 47935843 | 0.0023525 | 344292 | 0.000183451 |
| rs1445649 | -0.0014014 | 0.53787 | 2 | 155682556 | 0.0023347 | 344292 | 0.54836 |
| rs1555445 | 0.0034551 | 0.30934 | 20 | 31175258 | 0.0025288 | 344292 | 0.17185 |
| rs1565735 | -0.0051376 | 0.20143 | 8 | 27426077 | 0.0029219 | 344292 | 0.0786937 |
| rs1869243 | 0.0061126 | 0.46976 | 3 | 5724536 | 0.0023385 | 344292 | 0.00895159 |
| rs1899896 | 0.0051575 | 0.29755 | 8 | 93201036 | 0.0025533 | 344292 | 0.043395 |
| rs1971318 | -0.025964 | 0.15852 | 12 | 121389500 | 0.003192 | 344292 | 4.17E-16 |
| rs2046850 | -0.00029608 | 0.19443 | 1 | 210304319 | 0.0029513 | 344292 | 0.92009 |
| rs2050586 | -0.0042425 | 0.36007 | 1 | 87905828 | 0.0024337 | 344292 | 0.0812943 |
| rs2107300 | -0.0094515 | 0.84277 | 2 | 200937901 | 0.0032114 | 344292 | 0.00324997 |
| rs2140114 | -0.0048241 | 0.53668 | 7 | 3407568 | 0.0023355 | 344292 | 0.0388723 |
| rs2186122 | -0.0058667 | 0.5615 | 1 | 66470206 | 0.0023617 | 344292 | 0.0129879 |
| rs222449 | -0.00041902 | 0.80236 | 6 | 52916062 | 0.0029277 | 344292 | 0.88619 |
| rs2378662 | 0.0006889 | 0.54231 | 9 | 86707289 | 0.0023441 | 344292 | 0.768851 |
| rs240963 | 0.00047503 | 0.84066 | 6 | 111644332 | 0.0031846 | 344292 | 0.88142 |
| rs2631024 | -0.0034718 | 0.74021 | 8 | 91995577 | 0.0026645 | 344292 | 0.19258 |
| rs266047 | -0.0011995 | 0.53458 | 2 | 104088751 | 0.0023307 | 344292 | 0.606789 |
| rs3001723 | 0.0063668 | 0.29979 | 1 | 44037685 | 0.0025374 | 344292 | 0.0121001 |
| rs301805 | 0.0024728 | 0.58609 | 1 | 8481016 | 0.0023639 | 344292 | 0.29554 |
| rs35702515 | 2.08E-05 | 0.23544 | 2 | 137542847 | 0.0027688 | 344292 | 0.994 |
| rs3800227 | 0.0022005 | 0.74431 | 6 | 108994161 | 0.002675 | 344292 | 0.41073 |
| rs3801289 | 0.0068056 | 0.33794 | 7 | 96638267 | 0.0024677 | 344292 | 0.00581822 |
| rs3904512 | -0.0022375 | 0.45073 | 13 | 38357471 | 0.002342 | 344292 | 0.33938 |
| rs4044321 | -0.00391 | 0.6401 | 5 | 166989513 | 0.0024285 | 344292 | 0.10738 |
| rs4236259 | 0.0011569 | 0.49088 | 7 | 1708080 | 0.0023517 | 344292 | 0.62277 |
| rs4352629 | 0.00070544 | 0.45671 | 5 | 87756821 | 0.0023387 | 344292 | 0.762931 |
| rs4523689 | -0.0043186 | 0.3926 | 11 | 7950797 | 0.0023797 | 344292 | 0.0695633 |
| rs4543592 | -0.0029845 | 0.47993 | 9 | 3014254 | 0.0023326 | 344292 | 0.20072 |
| rs4674993 | 0.0053911 | 0.19892 | 2 | 226332033 | 0.0029159 | 344292 | 0.0644807 |
| rs4759228 | -0.002676 | 0.29702 | 12 | 56508409 | 0.002553 | 344292 | 0.29456 |
| rs4781977 | -0.0017141 | 0.22599 | 16 | 17572674 | 0.0028114 | 344292 | 0.54206 |
| rs4785836 | 0.0023617 | 0.37617 | 16 | 65604652 | 0.0024074 | 344292 | 0.32659 |
| rs578584 | 0.0068344 | 0.55836 | 2 | 45143175 | 0.0023472 | 344292 | 0.00359468 |
| rs6265 | -0.0064321 | 0.1887 | 11 | 27679916 | 0.0029727 | 344292 | 0.0304881 |
| rs6433897 | 0.013898 | 0.73637 | 2 | 182034448 | 0.0026446 | 344292 | 1.48E-07 |
| rs6508144 | 0.0021165 | 0.56994 | 18 | 50026142 | 0.0023567 | 344292 | 0.36915 |
| rs66680800 | -0.0046476 | 0.39963 | 3 | 85985324 | 0.0023745 | 344292 | 0.0503165 |
| rs6669839 | 0.0028643 | 0.20992 | 1 | 50625979 | 0.0028618 | 344292 | 0.31688 |
| rs6728726 | 0.006415 | 0.82767 | 2 | 623976 | 0.0030768 | 344292 | 0.0370749 |
| rs6788098 | 0.0055834 | 0.62823 | 3 | 85624131 | 0.0024057 | 344292 | 0.0202908 |
| rs6893752 | 0.0001545 | 0.74086 | 5 | 60374912 | 0.0026571 | 344292 | 0.95363 |
| rs7197072 | -0.0084235 | 0.22624 | 16 | 717085 | 0.0027851 | 344292 | 0.00249109 |
| rs7224742 | 0.0036659 | 0.61986 | 17 | 30657058 | 0.0024002 | 344292 | 0.12668 |
| rs72789632 | -0.0055162 | 0.13049 | 5 | 106834363 | 0.0034753 | 344292 | 0.11245 |
| rs72896886 | -0.0053767 | 0.16737 | 18 | 42632652 | 0.0031293 | 344292 | 0.0857591 |
| rs7322872 | -0.005556 | 0.79016 | 13 | 100548329 | 0.0028677 | 344292 | 0.0526914 |
| rs7555507 | -0.0034513 | 0.51462 | 1 | 73766037 | 0.0023312 | 344292 | 0.13874 |
| rs7585579 | -0.0054481 | 0.50974 | 2 | 60024857 | 0.0023514 | 344292 | 0.0205041 |
| rs76214862 | 0.0046163 | 0.18691 | 14 | 29500130 | 0.0029919 | 344292 | 0.12285 |
| rs76608582 | -0.0093418 | 0.047619 | 19 | 4474725 | 0.0057362 | 344292 | 0.10341 |
| rs7921378 | -0.0035765 | 0.48089 | 10 | 63674885 | 0.0023375 | 344292 | 0.12601 |
| rs7929518 | 0.0025469 | 0.78126 | 11 | 85980958 | 0.0028151 | 344292 | 0.3656 |
| rs7938812 | -0.0048376 | 0.38678 | 11 | 112911004 | 0.0023921 | 344292 | 0.043139 |
| rs7969559 | -0.0034249 | 0.72265 | 12 | 69655167 | 0.0026001 | 344292 | 0.18777 |
| rs9401770 | -0.0021445 | 0.26859 | 6 | 98748008 | 0.0026294 | 344292 | 0.41473 |
| rs9423279 | 0.0033886 | 0.65673 | 10 | 125680419 | 0.0024969 | 344292 | 0.17475 |
| rs9540729 | 0.0020817 | 0.52224 | 13 | 66947124 | 0.0023296 | 344292 | 0.37155 |
| rs962625 | 0.0025099 | 0.26855 | 4 | 28473524 | 0.0026359 | 344292 | 0.341 |
| rs9835772 | -0.0016519 | 0.24267 | 3 | 85766025 | 0.0027138 | 344292 | 0.54272 |
| rs993700 | -0.0098571 | 0.77769 | 4 | 67825894 | 0.0027986 | 344292 | 0.000428095 |

**Supplementary Table 12.** Detailed information on the SNPs for the MR analysis of smoking initiation and Alanine aminotransferase(ALT).

Removing the following SNPs for being palindromic with intermediate allele frequencies:

rs1160685, rs2186122, rs578584, rs6508144, rs7585579, rs7921378, rs9540729

| **SNP** | **beta.outcome** | **eaf.outcome** | **chr** | **pos** | **se.outcome** | **samplesize.outcome** | **pval.outcome** |
| --- | --- | --- | --- | --- | --- | --- | --- |
| rs10001365 | 0.00029808 | 0.38619 | 4 | 147797214 | 0.0022954 | 344136 | 0.89668 |
| rs10114490 | -0.001387 | 0.18553 | 9 | 11070165 | 0.0028736 | 344136 | 0.62934 |
| rs10159545 | -0.0004752 | 0.34791 | 10 | 21766969 | 0.0023635 | 344136 | 0.84065 |
| rs10233018 | 0.00086895 | 0.50411 | 7 | 117523709 | 0.0022373 | 344136 | 0.697729 |
| rs10260968 | -0.0011 | 0.58881 | 7 | 1889773 | 0.0022685 | 344136 | 0.62774 |
| rs10279261 | -0.0022955 | 0.61769 | 7 | 133589846 | 0.0023078 | 344136 | 0.31992 |
| rs10498846 | 0.002862 | 0.50754 | 6 | 67405337 | 0.0022477 | 344136 | 0.20292 |
| rs1050847 | -0.003276 | 0.57909 | 16 | 87443734 | 0.0022697 | 344136 | 0.14892 |
| rs10905461 | 2.92E-05 | 0.74428 | 10 | 8803551 | 0.0025636 | 344136 | 0.99091 |
| rs11057005 | -0.0012315 | 0.43785 | 12 | 16748721 | 0.0022661 | 344136 | 0.58683 |
| rs11078713 | -0.0061129 | 0.41765 | 17 | 7795972 | 0.0022685 | 344136 | 0.00704677 |
| rs1154693 | 0.0042873 | 0.85139 | 3 | 117804154 | 0.0031559 | 344136 | 0.17431 |
| rs1160685 | 0.00032427 | 0.44087 | 4 | 94052854 | 0.0022523 | 344136 | 0.88552 |
| rs11658881 | -0.008575 | 0.42027 | 17 | 2072949 | 0.0022668 | 344136 | 0.000155082 |
| rs11712680 | 0.00070224 | 0.18622 | 3 | 75009019 | 0.0028706 | 344136 | 0.80674 |
| rs117143374 | -0.021768 | 0.14186 | 21 | 40555561 | 0.0032115 | 344136 | 1.22E-11 |
| rs11872397 | -0.00055919 | 0.25979 | 18 | 72535282 | 0.0025783 | 344136 | 0.8283 |
| rs12025237 | -0.004333 | 0.12589 | 1 | 154205120 | 0.0033695 | 344136 | 0.19846 |
| rs12042107 | -0.0027577 | 0.54982 | 1 | 91196176 | 0.0022507 | 344136 | 0.22047 |
| rs12112638 | 0.0034653 | 0.26338 | 7 | 69735251 | 0.0025387 | 344136 | 0.17226 |
| rs12186738 | -0.0033137 | 0.14223 | 5 | 103816655 | 0.0031998 | 344136 | 0.3004 |
| rs12333760 | -0.00035737 | 0.16502 | 7 | 99185406 | 0.0030151 | 344136 | 0.90565 |
| rs12356821 | -0.0021032 | 0.14865 | 10 | 104563808 | 0.0031838 | 344136 | 0.50888 |
| rs12441907 | 0.0053444 | 0.18909 | 15 | 83922387 | 0.0028786 | 344136 | 0.0633665 |
| rs12474587 | 0.001016 | 0.45073 | 2 | 162802993 | 0.0022497 | 344136 | 0.65155 |
| rs12545053 | -0.0058522 | 0.40326 | 8 | 65073605 | 0.0022835 | 344136 | 0.010382 |
| rs12632110 | 0.001793 | 0.65822 | 3 | 50224225 | 0.0023611 | 344136 | 0.44763 |
| rs13030994 | -0.00015502 | 0.4823 | 2 | 146143090 | 0.0022363 | 344136 | 0.94474 |
| rs13145728 | -0.0035439 | 0.38202 | 4 | 140927812 | 0.0023022 | 344136 | 0.12373 |
| rs13261666 | -0.0021135 | 0.50398 | 8 | 59814666 | 0.0022342 | 344136 | 0.34416 |
| rs134529 | -0.015451 | 0.38242 | 22 | 28781758 | 0.0023021 | 344136 | 1.93E-11 |
| rs1385108 | 0.0010281 | 0.24248 | 5 | 154839646 | 0.0026149 | 344136 | 0.694201 |
| rs1435741 | -0.002984 | 0.43 | 15 | 47935843 | 0.0022588 | 344136 | 0.18648 |
| rs1445649 | 0.001484 | 0.53785 | 2 | 155682556 | 0.0022416 | 344136 | 0.50795 |
| rs1555445 | 0.0040833 | 0.30933 | 20 | 31175258 | 0.002428 | 344136 | 0.0926168 |
| rs1565735 | 0.0034205 | 0.2014 | 8 | 27426077 | 0.0028056 | 344136 | 0.22279 |
| rs1869243 | 0.0036088 | 0.4698 | 3 | 5724536 | 0.0022452 | 344136 | 0.10798 |
| rs1899896 | 0.0036527 | 0.29753 | 8 | 93201036 | 0.0024515 | 344136 | 0.13623 |
| rs1971318 | 0.0014824 | 0.15851 | 12 | 121389500 | 0.003065 | 344136 | 0.62863 |
| rs2046850 | -0.0020851 | 0.19443 | 1 | 210304319 | 0.0028336 | 344136 | 0.46184 |
| rs2050586 | 0.0011756 | 0.36006 | 1 | 87905828 | 0.0023367 | 344136 | 0.614889 |
| rs2107300 | -0.0029901 | 0.84279 | 2 | 200937901 | 0.0030836 | 344136 | 0.3322 |
| rs2140114 | -0.001152 | 0.53668 | 7 | 3407568 | 0.0022424 | 344136 | 0.607451 |
| rs2186122 | 0.0049196 | 0.56148 | 1 | 66470206 | 0.0022676 | 344136 | 0.0300421 |
| rs222449 | -0.0074705 | 0.80237 | 6 | 52916062 | 0.0028107 | 344136 | 0.0078643 |
| rs2378662 | -0.00089742 | 0.5423 | 9 | 86707289 | 0.0022507 | 344136 | 0.69009 |
| rs240963 | 0.0010482 | 0.84066 | 6 | 111644332 | 0.0030576 | 344136 | 0.73173 |
| rs2631024 | -0.0010013 | 0.74021 | 8 | 91995577 | 0.0025581 | 344136 | 0.69549 |
| rs266047 | -0.0059429 | 0.53458 | 2 | 104088751 | 0.0022378 | 344136 | 0.00791425 |
| rs3001723 | -0.0008491 | 0.2998 | 1 | 44037685 | 0.0024362 | 344136 | 0.72744 |
| rs301805 | 0.00028311 | 0.58608 | 1 | 8481016 | 0.0022696 | 344136 | 0.90073 |
| rs35702515 | 0.0023911 | 0.23541 | 2 | 137542847 | 0.0026585 | 344136 | 0.36843 |
| rs3800227 | 0.012006 | 0.74431 | 6 | 108994161 | 0.0025681 | 344136 | 2.94E-06 |
| rs3801289 | 0.00074396 | 0.33794 | 7 | 96638267 | 0.0023693 | 344136 | 0.75352 |
| rs3904512 | 0.0021951 | 0.45073 | 13 | 38357471 | 0.0022485 | 344136 | 0.32894 |
| rs4044321 | -0.0023074 | 0.64008 | 5 | 166989513 | 0.0023315 | 344136 | 0.32234 |
| rs4236259 | -0.0026379 | 0.49088 | 7 | 1708080 | 0.0022578 | 344136 | 0.24265 |
| rs4352629 | -0.00084396 | 0.45673 | 5 | 87756821 | 0.0022455 | 344136 | 0.70703 |
| rs4523689 | -0.0012363 | 0.39262 | 11 | 7950797 | 0.0022848 | 344136 | 0.588451 |
| rs4543592 | -0.00094501 | 0.47991 | 9 | 3014254 | 0.0022396 | 344136 | 0.673049 |
| rs4674993 | 0.0011185 | 0.19895 | 2 | 226332033 | 0.0027997 | 344136 | 0.68953 |
| rs4759228 | -0.013468 | 0.29703 | 12 | 56508409 | 0.0024511 | 344136 | 3.91E-08 |
| rs4781977 | 5.92E-05 | 0.22597 | 16 | 17572674 | 0.0026993 | 344136 | 0.9825 |
| rs4785836 | 0.00048246 | 0.37619 | 16 | 65604652 | 0.0023114 | 344136 | 0.83466 |
| rs578584 | 0.001488 | 0.55834 | 2 | 45143175 | 0.0022537 | 344136 | 0.5091 |
| rs6265 | -0.011604 | 0.18873 | 11 | 27679916 | 0.002854 | 344136 | 4.79E-05 |
| rs6433897 | 0.0066432 | 0.73636 | 2 | 182034448 | 0.0025391 | 344136 | 0.00888853 |
| rs6508144 | 3.37E-05 | 0.56995 | 18 | 50026142 | 0.0022628 | 344136 | 0.98813 |
| rs66680800 | -0.0062856 | 0.39962 | 3 | 85985324 | 0.0022798 | 344136 | 0.00583284 |
| rs6669839 | 0.003057 | 0.20991 | 1 | 50625979 | 0.0027477 | 344136 | 0.26589 |
| rs6728726 | 0.0051326 | 0.82767 | 2 | 623976 | 0.0029541 | 344136 | 0.0823057 |
| rs6788098 | 0.0011844 | 0.62822 | 3 | 85624131 | 0.0023098 | 344136 | 0.608129 |
| rs6893752 | 0.0028859 | 0.74086 | 5 | 60374912 | 0.0025512 | 344136 | 0.25798 |
| rs7197072 | 0.0025625 | 0.22624 | 16 | 717085 | 0.0026741 | 344136 | 0.33793 |
| rs7224742 | -0.0044168 | 0.61985 | 17 | 30657058 | 0.0023045 | 344136 | 0.0552955 |
| rs72789632 | -0.0034353 | 0.13048 | 5 | 106834363 | 0.003337 | 344136 | 0.30327 |
| rs72896886 | -0.0027924 | 0.16738 | 18 | 42632652 | 0.0030044 | 344136 | 0.35267 |
| rs7322872 | -0.0020698 | 0.79015 | 13 | 100548329 | 0.0027532 | 344136 | 0.45217 |
| rs7555507 | 0.0013164 | 0.51463 | 1 | 73766037 | 0.0022382 | 344136 | 0.55641 |
| rs7585579 | -0.0019892 | 0.50976 | 2 | 60024857 | 0.0022577 | 344136 | 0.37828 |
| rs76214862 | -0.0016273 | 0.18693 | 14 | 29500130 | 0.0028724 | 344136 | 0.571041 |
| rs76608582 | -0.015937 | 0.047607 | 19 | 4474725 | 0.0055081 | 344136 | 0.00381057 |
| rs7921378 | 0.0036204 | 0.48091 | 10 | 63674885 | 0.0022443 | 344136 | 0.10672 |
| rs7929518 | 0.0030567 | 0.78125 | 11 | 85980958 | 0.0027027 | 344136 | 0.25806 |
| rs7938812 | 0.00017559 | 0.38678 | 11 | 112911004 | 0.0022968 | 344136 | 0.93906 |
| rs7969559 | -0.01077 | 0.72262 | 12 | 69655167 | 0.0024963 | 344136 | 1.60E-05 |
| rs9401770 | -0.0059139 | 0.26859 | 6 | 98748008 | 0.0025245 | 344136 | 0.0191492 |
| rs9423279 | 0.0026282 | 0.65673 | 10 | 125680419 | 0.0023973 | 344136 | 0.27296 |
| rs9540729 | -0.0045366 | 0.52222 | 13 | 66947124 | 0.0022366 | 344136 | 0.0425305 |
| rs962625 | 0.0052171 | 0.26854 | 4 | 28473524 | 0.0025307 | 344136 | 0.0392582 |
| rs9835772 | 0.00029263 | 0.24268 | 3 | 85766025 | 0.0026055 | 344136 | 0.91058 |
| rs993700 | -0.0052127 | 0.7777 | 4 | 67825894 | 0.002687 | 344136 | 0.052389 |

**Supplementary Table 13.** Detailed information on the SNPs for the MR analysis of smoking initiation and Total bilirubin(TBIL).

Removing the following SNPs for being palindromic with intermediate allele frequencies:

rs1160685, rs2186122, rs578584, rs6508144, rs7585579, rs7921378, rs9540729

**.**

| **SNP** | **beta.outcome** | **eaf.outcome** | | **chr** | **pos** | **se.outcome** | **samplesize.outcome** | **pval.outcome** |
| --- | --- | --- | --- | --- | --- | --- | --- | --- |
| rs10001365 | 0.0057877 | | 0.38614 | 4 | 147797214 | 0.0023625 | 342829 | 0.0142929 |
| rs10114490 | -0.010423 | | 0.18553 | 9 | 11070165 | 0.0029578 | 342829 | 0.000425324 |
| rs10159545 | -0.0025214 | 0.3479 | | 10 | 21766969 | 0.0024329 | 342829 | 0.30003 |
| rs10233018 | 0.00085631 | 0.50414 | | 7 | 117523709 | 0.0023029 | 342829 | 0.710009 |
| rs10260968 | 0.0028583 | 0.58885 | | 7 | 1889773 | 0.002335 | 342829 | 0.22091 |
| rs10279261 | 0.0025664 | 0.61771 | | 7 | 133589846 | 0.0023754 | 342829 | 0.27998 |
| rs10498846 | 0.00032564 | 0.50755 | | 6 | 67405337 | 0.0023135 | 342829 | 0.88806 |
| rs1050847 | -0.00024385 | 0.57911 | | 16 | 87443734 | 0.0023362 | 342829 | 0.91687 |
| rs10905461 | 0.0094678 | 0.74424 | | 10 | 8803551 | 0.0026384 | 342829 | 0.000332637 |
| rs11057005 | 0.00080417 | 0.43784 | | 12 | 16748721 | 0.0023326 | 342829 | 0.730279 |
| rs11078713 | 0.014903 | 0.41769 | | 17 | 7795972 | 0.0023347 | 342829 | 1.74E-10 |
| rs1154693 | -0.00072358 | 0.85141 | | 3 | 117804154 | 0.0032484 | 342829 | 0.82373 |
| rs1160685 | 0.00056635 | 0.44084 | | 4 | 94052854 | 0.0023182 | 342829 | 0.80699 |
| rs11658881 | -0.0018989 | 0.42024 | | 17 | 2072949 | 0.002333 | 342829 | 0.41569 |
| rs11712680 | 0.0020412 | 0.18622 | | 3 | 75009019 | 0.0029545 | 342829 | 0.48965 |
| rs117143374 | 0.0016491 | 0.14188 | | 21 | 40555561 | 0.0033055 | 342829 | 0.61787 |
| rs11872397 | 0.0041392 | 0.25985 | | 18 | 72535282 | 0.0026534 | 342829 | 0.11876 |
| rs12025237 | -0.0070227 | 0.12582 | | 1 | 154205120 | 0.0034686 | 342829 | 0.0429062 |
| rs12042107 | 0.0024344 | 0.54981 | | 1 | 91196176 | 0.0023166 | 342829 | 0.29332 |
| rs12112638 | 0.0015548 | 0.26339 | | 7 | 69735251 | 0.0026127 | 342829 | 0.55179 |
| rs12186738 | 0.0020269 | 0.14224 | | 5 | 103816655 | 0.0032932 | 342829 | 0.53823 |
| rs12333760 | 0.0095025 | 0.16505 | | 7 | 99185406 | 0.0031031 | 342829 | 0.0021971 |
| rs12356821 | -6.88E-05 | 0.14865 | | 10 | 104563808 | 0.0032768 | 342829 | 0.98324 |
| rs12441907 | 0.0010142 | 0.18913 | | 15 | 83922387 | 0.0029624 | 342829 | 0.732089 |
| rs12474587 | -0.00039592 | 0.45075 | | 2 | 162802993 | 0.0023154 | 342829 | 0.86423 |
| rs12545053 | 0.0048372 | 0.40332 | | 8 | 65073605 | 0.0023505 | 342829 | 0.0395941 |
| rs12632110 | -0.0044222 | 0.65824 | | 3 | 50224225 | 0.0024303 | 342829 | 0.0688224 |
| rs13030994 | -5.67E-05 | 0.48231 | | 2 | 146143090 | 0.0023018 | 342829 | 0.98035 |
| rs13145728 | -3.85E-06 | 0.38206 | | 4 | 140927812 | 0.0023696 | 342829 | 0.9987 |
| rs13261666 | -0.0042464 | 0.50404 | | 8 | 59814666 | 0.0022995 | 342829 | 0.0647963 |
| rs134529 | -0.0090977 | 0.38242 | | 22 | 28781758 | 0.0023697 | 342829 | 0.00012345 |
| rs1385108 | -0.0025919 | 0.24242 | | 5 | 154839646 | 0.002692 | 342829 | 0.33563 |
| rs1435741 | -0.0011876 | 0.43003 | | 15 | 47935843 | 0.0023247 | 342829 | 0.60943 |
| rs1445649 | -0.0047753 | 0.53782 | | 2 | 155682556 | 0.0023074 | 342829 | 0.0384982 |
| rs1555445 | -0.0063686 | 0.30933 | | 20 | 31175258 | 0.0024992 | 342829 | 0.0108251 |
| rs1565735 | 0.0017376 | 0.2014 | | 8 | 27426077 | 0.0028877 | 342829 | 0.54735 |
| rs1869243 | -0.0032368 | 0.46977 | | 3 | 5724536 | 0.0023109 | 342829 | 0.16132 |
| rs1899896 | -0.0017166 | 0.29753 | | 8 | 93201036 | 0.0025234 | 342829 | 0.49634 |
| rs1971318 | 0.0033494 | 0.15855 | | 12 | 121389500 | 0.0031544 | 342829 | 0.28832 |
| rs2046850 | 0.00078874 | 0.19445 | | 1 | 210304319 | 0.0029168 | 342829 | 0.786839 |
| rs2050586 | 0.0034174 | 0.36005 | | 1 | 87905828 | 0.0024052 | 342829 | 0.15537 |
| rs2107300 | 0.0033208 | 0.84277 | | 2 | 200937901 | 0.0031737 | 342829 | 0.29539 |
| rs2140114 | 0.0051313 | 0.53667 | | 7 | 3407568 | 0.002308 | 342829 | 0.0262011 |
| rs2186122 | -0.00073317 | 0.56151 | | 1 | 66470206 | 0.0023338 | 342829 | 0.753409 |
| rs222449 | 0.0048686 | 0.8024 | | 6 | 52916062 | 0.0028931 | 342829 | 0.0924123 |
| rs2378662 | -0.0037794 | 0.54229 | | 9 | 86707289 | 0.0023163 | 342829 | 0.10274 |
| rs240963 | 0.017061 | 0.84065 | | 6 | 111644332 | 0.0031468 | 342829 | 5.91E-08 |
| rs2631024 | -0.0017684 | 0.74025 | | 8 | 91995577 | 0.0026332 | 342829 | 0.50186 |
| rs266047 | 0.0043193 | 0.5346 | | 2 | 104088751 | 0.0023031 | 342829 | 0.0607267 |
| rs3001723 | -0.0026565 | 0.29978 | | 1 | 44037685 | 0.0025075 | 342829 | 0.28939 |
| rs301805 | -0.0032501 | 0.58601 | | 1 | 8481016 | 0.002336 | 342829 | 0.16414 |
| rs35702515 | -0.0025588 | 0.23544 | | 2 | 137542847 | 0.0027361 | 342829 | 0.3497 |
| rs3800227 | -0.0028758 | 0.74428 | | 6 | 108994161 | 0.0026434 | 342829 | 0.27664 |
| rs3801289 | -0.00079848 | 0.33797 | | 7 | 96638267 | 0.0024385 | 342829 | 0.743331 |
| rs3904512 | 0.0023863 | 0.45071 | | 13 | 38357471 | 0.0023143 | 342829 | 0.30249 |
| rs4044321 | 0.0059311 | 0.64012 | | 5 | 166989513 | 0.0023997 | 342829 | 0.013453 |
| rs4236259 | 0.0051897 | 0.49087 | | 7 | 1708080 | 0.0023237 | 342829 | 0.0255252 |
| rs4352629 | 0.0062591 | 0.4567 | | 5 | 87756821 | 0.0023113 | 342829 | 0.00676706 |
| rs4523689 | -2.93E-05 | 0.39255 | | 11 | 7950797 | 0.0023518 | 342829 | 0.99006 |
| rs4543592 | -0.00014722 | 0.47988 | | 9 | 3014254 | 0.0023051 | 342829 | 0.94908 |
| rs4674993 | 0.0027522 | 0.19895 | | 2 | 226332033 | 0.0028813 | 342829 | 0.33948 |
| rs4759228 | -0.00043217 | 0.29703 | | 12 | 56508409 | 0.0025231 | 342829 | 0.864 |
| rs4781977 | -0.00030925 | 0.22601 | | 16 | 17572674 | 0.0027782 | 342829 | 0.91137 |
| rs4785836 | 0.00026257 | 0.37621 | | 16 | 65604652 | 0.002379 | 342829 | 0.91212 |
| rs578584 | -0.00028183 | 0.55832 | | 2 | 45143175 | 0.0023194 | 342829 | 0.90329 |
| rs6265 | 0.0072221 | 0.18874 | | 11 | 27679916 | 0.0029374 | 342829 | 0.0139441 |
| rs6433897 | -0.0022248 | 0.73641 | | 2 | 182034448 | 0.0026133 | 342829 | 0.39458 |
| rs6508144 | -0.0019176 | 0.56999 | | 18 | 50026142 | 0.0023289 | 342829 | 0.41029 |
| rs66680800 | -0.0011104 | 0.39964 | | 3 | 85985324 | 0.0023466 | 342829 | 0.63606 |
| rs6669839 | -0.0013857 | 0.20988 | | 1 | 50625979 | 0.0028281 | 342829 | 0.62415 |
| rs6728726 | -0.0033237 | 0.82764 | | 2 | 623976 | 0.0030404 | 342829 | 0.27432 |
| rs6788098 | -0.00078024 | 0.62823 | | 3 | 85624131 | 0.0023775 | 342829 | 0.74278 |
| rs6893752 | 0.0066741 | 0.7409 | | 5 | 60374912 | 0.0026258 | 342829 | 0.0110301 |
| rs7197072 | 0.0069002 | 0.22622 | | 16 | 717085 | 0.0027525 | 342829 | 0.012179 |
| rs7224742 | 0.0046554 | 0.61992 | | 17 | 30657058 | 0.0023717 | 342829 | 0.0496581 |
| rs72789632 | 0.003609 | 0.13049 | | 5 | 106834363 | 0.0034342 | 342829 | 0.29331 |
| rs72896886 | 0.0025036 | 0.16733 | | 18 | 42632652 | 0.0030924 | 342829 | 0.41817 |
| rs7322872 | 0.008481 | 0.79018 | | 13 | 100548329 | 0.0028341 | 342829 | 0.00276707 |
| rs7555507 | 0.0011098 | 0.51459 | | 1 | 73766037 | 0.0023038 | 342829 | 0.630001 |
| rs7585579 | -9.55E-05 | 0.50977 | | 2 | 60024857 | 0.0023239 | 342829 | 0.96724 |
| rs76214862 | 0.00097112 | 0.18692 | | 14 | 29500130 | 0.0029566 | 342829 | 0.742569 |
| rs76608582 | 0.0097273 | 0.047624 | | 19 | 4474725 | 0.0056674 | 342829 | 0.0860994 |
| rs7921378 | -0.0057188 | 0.48097 | | 10 | 63674885 | 0.0023099 | 342829 | 0.013296 |
| rs7929518 | 0.00095435 | 0.78121 | | 11 | 85980958 | 0.0027815 | 342829 | 0.73152 |
| rs7938812 | -0.0021317 | 0.38675 | | 11 | 112911004 | 0.0023641 | 342829 | 0.36723 |
| rs7969559 | 0.0019072 | 0.72264 | | 12 | 69655167 | 0.0025692 | 342829 | 0.4579 |
| rs9401770 | 0.00095393 | 0.26864 | | 6 | 98748008 | 0.0025981 | 342829 | 0.71349 |
| rs9423279 | -0.0016046 | 0.65673 | | 10 | 125680419 | 0.0024673 | 342829 | 0.515451 |
| rs9540729 | 0.0024314 | 0.52223 | | 13 | 66947124 | 0.0023021 | 342829 | 0.29088 |
| rs962625 | -0.0011673 | 0.2685 | | 4 | 28473524 | 0.002605 | 342829 | 0.65408 |
| rs9835772 | -0.0010166 | 0.2427 | | 3 | 85766025 | 0.0026815 | 342829 | 0.70461 |
| rs993700 | 0.009481 | 0.77773 | | 4 | 67825894 | 0.0027658 | 342829 | 0.000608163 |

**Supplementary Table 14.** Detailed information on the SNPs for the MR analysis of smoking initiation and albumin(ALB).

Removing the following SNPs for being palindromic with intermediate allele frequencies:

rs1160685, rs2186122, rs578584, rs6508144, rs7585579, rs7921378, rs9540729

|  |  |  |  |  |  | |  | |  |
| --- | --- | --- | --- | --- | --- | --- | --- | --- | --- |
| **SNP** | **beta.outcome** | **eaf.outcome** | **chr** | **Pos** | **se.outcome** | **samplesize.**  **outcome** | | | **pval.outcome** |
| rs10001365 | -0.0010539 | 0.38616 | 4 | 147797214 | 0.0025282 | | 315268 | | 0.67679 |
| rs10114490 | 0.001918 | 0.18568 | 9 | 11070165 | 0.0031638 | | 315268 | | 0.54436 |
| rs10159545 | -0.019222 | 0.34789 | 10 | 21766969 | 0.0026053 | | 315268 | | 1.61E-13 |
| rs10233018 | -0.0023898 | 0.50402 | 7 | 117523709 | 0.002465 | | 315268 | | 0.3323 |
| rs10260968 | 0.0085725 | 0.58906 | 7 | 1889773 | 0.0024999 | | 315268 | | 0.000605662 |
| rs10279261 | -0.0021462 | 0.61776 | 7 | 133589846 | 0.0025433 | | 315268 | | 0.39874 |
| rs10498846 | -0.0049802 | 0.5073 | 6 | 67405337 | 0.0024766 | | 315268 | | 0.0443353 |
| rs1050847 | 0.0055415 | 0.5791 | 16 | 87443734 | 0.0025002 | | 315268 | | 0.0266649 |
| rs10905461 | 0.0040469 | 0.74407 | 10 | 8803551 | 0.002823 | | 315268 | | 0.1517 |
| rs11057005 | -0.00073925 | 0.43763 | 12 | 16748721 | 0.002497 | | 315268 | | 0.76719 |
| rs11078713 | -0.0011903 | 0.4177 | 17 | 7795972 | 0.0024991 | | 315268 | | 0.633859 |
| rs1154693 | -0.00044931 | 0.85127 | 3 | 117804154 | 0.0034756 | | 315268 | | 0.89714 |
| rs1160685 | -0.0010456 | 0.44086 | 4 | 94052854 | 0.0024808 | | 315268 | | 0.673429 |
| rs11658881 | -0.011389 | 0.42026 | 17 | 2072949 | 0.0024976 | | 315268 | | 5.12E-06 |
| rs11712680 | -0.00074999 | 0.18628 | 3 | 75009019 | 0.0031616 | | 315268 | | 0.81249 |
| rs117143374 | -0.009542 | 0.14191 | 21 | 40555561 | 0.003536 | | 315268 | | 0.00696482 |
| rs11872397 | 0.0021591 | 0.25974 | 18 | 72535282 | 0.0028415 | | 315268 | | 0.44736 |
| rs12025237 | -0.0029475 | 0.12569 | 1 | 154205120 | 0.0037161 | | 315268 | | 0.42769 |
| rs12042107 | 0.0067106 | 0.54961 | 1 | 91196176 | 0.0024785 | | 315268 | | 0.00677782 |
| rs12112638 | -0.0056639 | 0.26326 | 7 | 69735251 | 0.0027978 | | 315268 | | 0.0429309 |
| rs12186738 | 0.0021555 | 0.14236 | 5 | 103816655 | 0.0035239 | | 315268 | | 0.540741 |
| rs12333760 | -0.00079988 | 0.16509 | 7 | 99185406 | 0.0033216 | | 315268 | | 0.8097 |
| rs12356821 | -0.0075551 | 0.14871 | 10 | 104563808 | 0.0035071 | | 315268 | | 0.0312219 |
| rs12441907 | -0.0056296 | 0.18906 | 15 | 83922387 | 0.0031709 | | 315268 | | 0.0758368 |
| rs12474587 | 0.0047695 | 0.45078 | 2 | 162802993 | 0.0024786 | | | 315268 | 0.0543238 |
| rs12545053 | 0.0034644 | 0.40338 | 8 | 65073605 | 0.0025162 | | 315268 | | 0.16856 |
| rs12632110 | 0.002728 | 0.65788 | 3 | 50224225 | 0.0026013 | | 315268 | | 0.29432 |
| rs13030994 | 0.0015448 | 0.48225 | 2 | 146143090 | 0.0024639 | | 315268 | | 0.53066 |
| rs13145728 | 0.0030052 | 0.38209 | 4 | 140927812 | 0.002536 | | 315268 | | 0.23602 |
| rs13261666 | 0.0014866 | 0.50378 | 8 | 59814666 | 0.0024624 | | 315268 | | 0.546011 |
| rs134529 | 0.0038969 | 0.38229 | 22 | 28781758 | 0.0025363 | | 315268 | | 0.12443 |
| rs1385108 | 0.00034496 | 0.24247 | 5 | 154839646 | 0.0028814 | | 315268 | | 0.90471 |
| rs1435741 | -0.0014431 | 0.42979 | 15 | 47935843 | 0.0024879 | | 315268 | | 0.561881 |
| rs1445649 | -0.0049711 | 0.53771 | 2 | 155682556 | 0.0024688 | | 315268 | | 0.0440565 |
| rs1555445 | -0.0070231 | 0.30943 | 20 | 31175258 | 0.0026744 | | 315268 | | 0.00863754 |
| rs1565735 | 0.0017523 | 0.20158 | 8 | 27426077 | 0.0030892 | | 315268 | | 0.57056 |
| rs1869243 | 0.0010222 | 0.46962 | 3 | 5724536 | 0.0024728 | | 315268 | | 0.679319 |
| rs1899896 | -0.0031189 | 0.29757 | 8 | 93201036 | 0.0027011 | | 315268 | | 0.24823 |
| rs1971318 | 0.001576 | 0.15857 | 12 | 121389500 | 0.0033768 | | 315268 | | 0.6407 |
| rs2046850 | -0.0043584 | 0.19451 | 1 | 210304319 | 0.0031209 | | 315268 | | 0.16256 |
| rs2050586 | 0.0014465 | 0.36009 | 1 | 87905828 | 0.0025737 | | 315268 | | 0.57411 |
| rs2107300 | -0.0011907 | 0.8427 | 2 | 200937901 | 0.0033967 | | 315268 | | 0.72593 |
| rs2140114 | -0.0022442 | 0.53686 | 7 | 3407568 | 0.0024705 | | 315268 | | 0.36366 |
| rs2186122 | 0.0084107 | 0.56143 | 1 | 66470206 | 0.0024981 | | 315268 | | 0.000760396 |
| rs222449 | 0.0065373 | 0.80208 | 6 | 52916062 | 0.0030953 | | 315268 | | 0.0346849 |
| rs2378662 | -0.0025368 | 0.54227 | 9 | 86707289 | 0.0024795 | | 315268 | | 0.30625 |
| rs240963 | 0.014845 | 0.84059 | 6 | 111644332 | 0.0033689 | | 315268 | | 1.05E-05 |
| rs2631024 | -0.00099482 | 0.7402 | 8 | 91995577 | 0.0028191 | | 315268 | | 0.724171 |
| rs266047 | 0.00026264 | 0.53449 | 2 | 104088751 | 0.0024655 | | 315268 | | 0.91517 |
| rs3001723 | -0.0048876 | 0.29982 | 1 | 44037685 | 0.0026839 | | 315268 | | 0.0685915 |
| rs301805 | -0.01027 | 0.58607 | 1 | 8481016 | 0.0025009 | | 315268 | | 4.02E-05 |
| rs35702515 | -0.0065396 | 0.2355 | 2 | 137542847 | 0.0029285 | | 315268 | | 0.0255452 |
| rs3800227 | -0.0046273 | 0.7441 | 6 | 108994161 | 0.0028277 | | 315268 | | 0.10175 |
| rs3801289 | 0.0045458 | 0.33784 | 7 | 96638267 | 0.0026099 | | 315268 | | 0.0815493 |
| rs3904512 | 0.0014547 | 0.4506 | 13 | 38357471 | 0.0024772 | | 315268 | | 0.55705 |
| rs4044321 | 0.0036109 | 0.64038 | 5 | 166989513 | 0.0025702 | | 315268 | | 0.16004 |
| rs4236259 | 0.0012471 | 0.49072 | 7 | 1708080 | 0.0024877 | | 315268 | | 0.616161 |
| rs4352629 | 0.0044499 | 0.45693 | 5 | 87756821 | 0.0024741 | | 315268 | | 0.0720825 |
| rs4523689 | 0.0024423 | 0.39267 | 11 | 7950797 | 0.0025168 | | 315268 | | 0.33185 |
| rs4543592 | 0.0055181 | 0.47967 | 9 | 3014254 | 0.002468 | | 315268 | | 0.0253647 |
| rs4674993 | 0.0021687 | 0.19889 | 2 | 226332033 | 0.003085 | | 315268 | | 0.48206 |
| rs4759228 | 0.0046326 | 0.29711 | 12 | 56508409 | 0.0027009 | | 315268 | | 0.0863118 |
| rs4781977 | 0.013238 | 0.22612 | 16 | 17572674 | 0.0029723 | | 315268 | | 8.44E-06 |
| rs4785836 | 0.0018531 | 0.37619 | 16 | 65604652 | 0.0025464 | | 315268 | | 0.46677 |
| rs578584 | -0.0023391 | 0.55861 | 2 | 45143175 | 0.0024831 | | 315268 | | 0.34618 |
| rs6265 | 0.0080658 | 0.18869 | 11 | 27679916 | 0.0031451 | | 315268 | | 0.0103309 |
| rs6433897 | 0.0019281 | 0.73646 | 2 | 182034448 | 0.0027974 | | 315268 | | 0.490669 |
| rs6508144 | -0.0019324 | 0.5698 | 18 | 50026142 | 0.0024923 | | 315268 | | 0.43814 |
| rs66680800 | -0.0045623 | 0.39985 | 3 | 85985324 | 0.0025115 | | 315268 | | 0.0692899 |
| rs6669839 | 0.0021744 | 0.2097 | 1 | 50625979 | 0.0030286 | | 315268 | | 0.47279 |
| rs6728726 | -0.0094119 | 0.82778 | 2 | 623976 | 0.0032542 | | 315268 | | 0.00382534 |
| rs6788098 | 0.0068094 | 0.62864 | 3 | 85624131 | 0.002545 | | 315268 | | 0.00746088 |
| rs6893752 | 0.0008592 | 0.74091 | 5 | 60374912 | 0.0028105 | | 315268 | | 0.75982 |
| rs7197072 | -0.0080147 | 0.22616 | 16 | 717085 | 0.0029475 | | 315268 | | 0.00654531 |
| rs7224742 | -0.0057788 | 0.61974 | 17 | 30657058 | 0.0025385 | | 315268 | | 0.0228192 |
| rs72789632 | -0.0032017 | 0.13052 | 5 | 106834363 | 0.0036756 | | 315268 | | 0.38371 |
| rs72896886 | -0.0020742 | 0.16728 | 18 | 42632652 | 0.0033105 | | 315268 | | 0.53097 |
| rs7322872 | 0.0078825 | 0.79027 | 13 | 100548329 | 0.0030329 | | 315268 | | 0.00934953 |
| rs7555507 | 0.0045256 | 0.51468 | 1 | 73766037 | 0.0024662 | | 315268 | | 0.0664967 |
| rs7585579 | 0.0033229 | 0.51001 | 2 | 60024857 | 0.002488 | | 315268 | | 0.18168 |
| rs76214862 | 0.0012442 | 0.18673 | 14 | 29500130 | 0.0031661 | | 315268 | | 0.69433 |
| rs76608582 | 0.015222 | 0.04755 | 19 | 4474725 | 0.0060748 | | 315268 | | 0.0122169 |
| rs7921378 | 0.0075175 | 0.48065 | 10 | 63674885 | 0.0024721 | | 315268 | | 0.00235858 |
| rs7929518 | 0.0048167 | 0.78113 | 11 | 85980958 | 0.0029765 | | 315268 | | 0.10561 |
| rs7938812 | 0.0048404 | 0.38708 | 11 | 112911004 | 0.00253 | | 315268 | | 0.0557186 |
| rs7969559 | -0.0058241 | 0.72286 | 12 | 69655167 | 0.0027503 | | 315268 | | 0.0342098 |
| rs9401770 | -0.0058766 | 0.26877 | 6 | 98748008 | 0.0027796 | | 315268 | | 0.0345017 |
| rs9423279 | -0.0012143 | 0.6565 | 10 | 125680419 | 0.0026414 | | 315268 | | 0.64572 |
| rs9540729 | 0.00099595 | 0.52252 | 13 | 66947124 | 0.0024636 | | 315268 | | 0.68602 |
| rs962625 | -0.0018423 | 0.2686 | 4 | 28473524 | 0.0027866 | | 315268 | | 0.50852 |
| rs9835772 | -0.0028288 | 0.24242 | 3 | 85766025 | 0.0028723 | | 315268 | | 0.32469 |
| rs993700 | 0.0028867 | 0.77765 | 4 | 67825894 | 0.0029608 | | 315268 | | 0.32957 |

**Supplementary Table 15.** Detailed information on the SNPs for the MR analysis of smoking initiation and Aspartate aminotransferase(AST).

Removing the following SNPs for being palindromic with intermediate allele frequencies:

rs1160685, rs2186122, rs578584, rs6508144, rs7585579, rs7921378, rs9540729

| **SNP** | **beta.outcome** | **eaf.outcome** | **chr** | **pos** | **se.outcome** | **samplesize.outcome** | **pval.outcome** |
| --- | --- | --- | --- | --- | --- | --- | --- |
| rs10001365 | 0.0025117 | 0.38614 | 4 | 147797214 | 0.0023497 | 342990 | 0.2851 |
| rs10114490 | -0.0018747 | 0.18552 | 9 | 11070165 | 0.002942 | 342990 | 0.52399 |
| rs10159545 | -0.010294 | 0.34788 | 10 | 21766969 | 0.0024197 | 342990 | 2.10E-05 |
| rs10233018 | 0.00055192 | 0.50413 | 7 | 117523709 | 0.0022904 | 342990 | 0.80958 |
| rs10260968 | -0.00057106 | 0.58885 | 7 | 1889773 | 0.0023223 | 342990 | 0.80576 |
| rs10279261 | -0.0036102 | 0.61771 | 7 | 133589846 | 0.0023627 | 342990 | 0.12651 |
| rs10498846 | 0.0025506 | 0.50753 | 6 | 67405337 | 0.002301 | 342990 | 0.26766 |
| rs1050847 | -0.0029675 | 0.57912 | 16 | 87443734 | 0.0023234 | 342990 | 0.20153 |
| rs10905461 | 0.00081164 | 0.74423 | 10 | 8803551 | 0.0026243 | 342990 | 0.75711 |
| rs11057005 | 0.0002069 | 0.43789 | 12 | 16748721 | 0.00232 | 342990 | 0.92894 |
| rs11078713 | -0.0023017 | 0.41764 | 17 | 7795972 | 0.0023223 | 342990 | 0.32162 |
| rs1154693 | 6.77E-06 | 0.85141 | 3 | 117804154 | 0.0032309 | 342990 | 0.99833 |
| rs1160685 | 0.00089328 | 0.44083 | 4 | 94052854 | 0.0023057 | 342990 | 0.698449 |
| rs11658881 | -0.013723 | 0.42024 | 17 | 2072949 | 0.0023204 | 342990 | 3.34E-09 |
| rs11712680 | 0.0021605 | 0.18622 | 3 | 75009019 | 0.0029386 | 342990 | 0.46221 |
| rs117143374 | -0.0071327 | 0.14186 | 21 | 40555561 | 0.0032879 | 342990 | 0.0300511 |
| rs11872397 | -0.0013863 | 0.25985 | 18 | 72535282 | 0.0026391 | 342990 | 0.599389 |
| rs12025237 | 0.0021492 | 0.12583 | 1 | 154205120 | 0.0034497 | 342990 | 0.53328 |
| rs12042107 | -0.0013189 | 0.5498 | 1 | 91196176 | 0.0023041 | 342990 | 0.56704 |
| rs12112638 | -0.0054038 | 0.26339 | 7 | 69735251 | 0.0025987 | 342990 | 0.0375803 |
| rs12186738 | 0.002409 | 0.14226 | 5 | 103816655 | 0.0032753 | 342990 | 0.46202 |
| rs12333760 | 0.0049756 | 0.16506 | 7 | 99185406 | 0.0030864 | 342990 | 0.10693 |
| rs12356821 | 0.004325 | 0.14866 | 10 | 104563808 | 0.003259 | 342990 | 0.18448 |
| rs12441907 | 0.0012165 | 0.18909 | 15 | 83922387 | 0.0029467 | 342990 | 0.679729 |
| rs12474587 | 0.0018915 | 0.45075 | 2 | 162802993 | 0.002303 | 342990 | 0.41146 |
| rs12545053 | -0.0030784 | 0.4033 | 8 | 65073605 | 0.0023377 | 342990 | 0.18788 |
| rs12632110 | -0.0019653 | 0.65824 | 3 | 50224225 | 0.0024172 | 342990 | 0.41619 |
| rs13030994 | -0.0061196 | 0.48232 | 2 | 146143090 | 0.0022893 | 342990 | 0.00751571 |
| rs13145728 | 0.0009623 | 0.38205 | 4 | 140927812 | 0.0023569 | 342990 | 0.68306 |
| rs13261666 | 0.0048847 | 0.50403 | 8 | 59814666 | 0.0022871 | 342990 | 0.0327009 |
| rs134529 | -0.013248 | 0.38238 | 22 | 28781758 | 0.0023568 | 342990 | 1.90E-08 |
| rs1385108 | 0.0034852 | 0.24245 | 5 | 154839646 | 0.0026771 | 342990 | 0.19297 |
| rs1435741 | -0.0024844 | 0.43004 | 15 | 47935843 | 0.0023121 | 342990 | 0.28259 |
| rs1445649 | -0.0016897 | 0.53781 | 2 | 155682556 | 0.002295 | 342990 | 0.46157 |
| rs1555445 | 0.0055022 | 0.30933 | 20 | 31175258 | 0.0024857 | 342990 | 0.0268621 |
| rs1565735 | 0.0042731 | 0.20139 | 8 | 27426077 | 0.0028721 | 342990 | 0.13681 |
| rs1869243 | 0.0022221 | 0.46977 | 3 | 5724536 | 0.0022984 | 342990 | 0.33365 |
| rs1899896 | -0.00062233 | 0.29751 | 8 | 93201036 | 0.0025098 | 342990 | 0.80416 |
| rs1971318 | 0.00072766 | 0.15855 | 12 | 121389500 | 0.0031375 | 342990 | 0.8166 |
| rs2046850 | 0.00022566 | 0.19447 | 1 | 210304319 | 0.0029011 | 342990 | 0.938 |
| rs2050586 | 0.0044154 | 0.36006 | 1 | 87905828 | 0.0023922 | 342990 | 0.0649292 |
| rs2107300 | 4.67E-05 | 0.84276 | 2 | 200937901 | 0.0031564 | 342990 | 0.9882 |
| rs2140114 | -0.001805 | 0.53668 | 7 | 3407568 | 0.0022955 | 342990 | 0.4317 |
| rs2186122 | 0.0054345 | 0.56148 | 1 | 66470206 | 0.0023212 | 342990 | 0.0192229 |
| rs222449 | -0.00087323 | 0.80238 | 6 | 52916062 | 0.0028772 | 342990 | 0.761511 |
| rs2378662 | -0.0022162 | 0.5423 | 9 | 86707289 | 0.0023039 | 342990 | 0.33606 |
| rs240963 | 0.004846 | 0.84067 | 6 | 111644332 | 0.0031301 | 342990 | 0.12158 |
| rs2631024 | 0.0018344 | 0.74023 | 8 | 91995577 | 0.0026189 | 342990 | 0.48364 |
| rs266047 | -0.0022453 | 0.53461 | 2 | 104088751 | 0.0022908 | 342990 | 0.32701 |
| rs3001723 | 0.0028247 | 0.29979 | 1 | 44037685 | 0.0024938 | 342990 | 0.25734 |
| rs301805 | -0.00088531 | 0.58602 | 1 | 8481016 | 0.0023235 | 342990 | 0.703191 |
| rs35702515 | 0.0039462 | 0.23542 | 2 | 137542847 | 0.0027215 | 342990 | 0.14705 |
| rs3800227 | 0.0039577 | 0.74428 | 6 | 108994161 | 0.0026291 | 342990 | 0.13224 |
| rs3801289 | 0.00076477 | 0.33798 | 7 | 96638267 | 0.0024252 | 342990 | 0.752501 |
| rs3904512 | 0.0015003 | 0.45071 | 13 | 38357471 | 0.0023019 | 342990 | 0.51454 |
| rs4044321 | 0.0031999 | 0.64009 | 5 | 166989513 | 0.0023867 | 342990 | 0.18002 |
| rs4236259 | -0.0017128 | 0.49086 | 7 | 1708080 | 0.0023112 | 342990 | 0.45866 |
| rs4352629 | -0.0015642 | 0.45671 | 5 | 87756821 | 0.0022988 | 342990 | 0.49622 |
| rs4523689 | -0.0028386 | 0.39257 | 11 | 7950797 | 0.0023391 | 342990 | 0.22491 |
| rs4543592 | 0.0014447 | 0.47989 | 9 | 3014254 | 0.0022927 | 342990 | 0.5286 |
| rs4674993 | 0.0053866 | 0.19895 | 2 | 226332033 | 0.0028656 | 342990 | 0.0601465 |
| rs4759228 | -0.010952 | 0.29702 | 12 | 56508409 | 0.0025093 | 342990 | 1.27E-05 |
| rs4781977 | -0.0034625 | 0.22599 | 16 | 17572674 | 0.0027633 | 342990 | 0.21019 |
| rs4785836 | -0.00097866 | 0.37619 | 16 | 65604652 | 0.0023661 | 342990 | 0.67916 |
| rs578584 | 0.0027453 | 0.55833 | 2 | 45143175 | 0.002307 | 342990 | 0.23405 |
| rs6265 | -0.014683 | 0.18872 | 11 | 27679916 | 0.0029216 | 342990 | 5.02E-07 |
| rs6433897 | 0.001508 | 0.73641 | 2 | 182034448 | 0.0025994 | 342990 | 0.561809 |
| rs6508144 | -0.0009297 | 0.57001 | 18 | 50026142 | 0.0023164 | 342990 | 0.688159 |
| rs66680800 | -0.0024406 | 0.39963 | 3 | 85985324 | 0.0023338 | 342990 | 0.29567 |
| rs6669839 | 0.0010266 | 0.20991 | 1 | 50625979 | 0.0028126 | 342990 | 0.71512 |
| rs6728726 | 0.0073538 | 0.82765 | 2 | 623976 | 0.003024 | 342990 | 0.0150248 |
| rs6788098 | 0.00079668 | 0.62823 | 3 | 85624131 | 0.0023647 | 342990 | 0.73619 |
| rs6893752 | 0.0067431 | 0.74089 | 5 | 60374912 | 0.0026115 | 342990 | 0.00982132 |
| rs7197072 | 0.0020507 | 0.22622 | 16 | 717085 | 0.0027376 | 342990 | 0.45381 |
| rs7224742 | -0.0018708 | 0.61988 | 17 | 30657058 | 0.0023589 | 342990 | 0.42774 |
| rs72789632 | -0.002408 | 0.13049 | 5 | 106834363 | 0.0034158 | 342990 | 0.48083 |
| rs72896886 | -0.0023199 | 0.16733 | 18 | 42632652 | 0.0030757 | 342990 | 0.4507 |
| rs7322872 | -0.0032033 | 0.79015 | 13 | 100548329 | 0.0028186 | 342990 | 0.25577 |
| rs7555507 | 0.00097057 | 0.5146 | 1 | 73766037 | 0.0022914 | 342990 | 0.67188 |
| rs7585579 | -0.00053636 | 0.50977 | 2 | 60024857 | 0.0023114 | 342990 | 0.8165 |
| rs76214862 | 0.00065617 | 0.18694 | 14 | 29500130 | 0.0029405 | 342990 | 0.82342 |
| rs76608582 | -0.0029979 | 0.047622 | 19 | 4474725 | 0.0056371 | 342990 | 0.594849 |
| rs7921378 | 0.004581 | 0.48094 | 10 | 63674885 | 0.0022975 | 342990 | 0.0461594 |
| rs7929518 | 0.00087882 | 0.78122 | 11 | 85980958 | 0.0027666 | 342990 | 0.750749 |
| rs7938812 | -0.0010158 | 0.38675 | 11 | 112911004 | 0.0023514 | 342990 | 0.66573 |
| rs7969559 | -0.01172 | 0.72262 | 12 | 69655167 | 0.0025553 | 342990 | 4.51E-06 |
| rs9401770 | -0.0022288 | 0.26864 | 6 | 98748008 | 0.002584 | 342990 | 0.38839 |
| rs9423279 | 0.00026226 | 0.65674 | 10 | 125680419 | 0.002454 | 342990 | 0.91489 |
| rs9540729 | 0.00027227 | 0.52223 | 13 | 66947124 | 0.0022896 | 342990 | 0.90534 |
| rs962625 | 0.004176 | 0.2685 | 4 | 28473524 | 0.0025909 | 342990 | 0.107 |
| rs9835772 | -0.0015092 | 0.24268 | 3 | 85766025 | 0.0026671 | 342990 | 0.57149 |
| rs993700 | -0.0015252 | 0.77776 | 4 | 67825894 | 0.002751 | 342990 | 0.579281 |

**Supplementary Table 16.** Detailed information on the SNPs for the MR analysis of smoking initiation and gamma glutamyltransferase(GGT).

Removing the following SNPs for being palindromic with intermediate allele frequencies:

rs1160685, rs2186122, rs578584, rs6508144, rs7585579, rs7921378, rs9540729

| **SNP** | **beta.outcome** | **eaf.outcome** | **chr** | **pos** | **se.outcome** | **samplesize.outcome** | **pval.outcome** |
| --- | --- | --- | --- | --- | --- | --- | --- |
| rs10001365 | -0.00096363 | 0.38616 | 4 | 147797214 | 0.002281 | 344104 | 0.67269 |
| rs10114490 | -0.0018163 | 0.18553 | 9 | 11070165 | 0.0028557 | 344104 | 0.52476 |
| rs10159545 | 0.0049335 | 0.34792 | 10 | 21766969 | 0.0023486 | 344104 | 0.0356788 |
| rs10233018 | 0.0076316 | 0.50412 | 7 | 117523709 | 0.0022233 | 344104 | 0.00059815 |
| rs10260968 | -0.00014202 | 0.58882 | 7 | 1889773 | 0.0022542 | 344104 | 0.94977 |
| rs10279261 | 0.0019214 | 0.6177 | 7 | 133589846 | 0.0022935 | 344104 | 0.40218 |
| rs10498846 | 0.0041534 | 0.50751 | 6 | 67405337 | 0.0022336 | 344104 | 0.062955 |
| rs1050847 | -0.0024952 | 0.57914 | 16 | 87443734 | 0.0022554 | 344104 | 0.26859 |
| rs10905461 | 0.00022657 | 0.74427 | 10 | 8803551 | 0.0025475 | 344104 | 0.92913 |
| rs11057005 | -0.0049375 | 0.43785 | 12 | 16748721 | 0.0022519 | 344104 | 0.0283341 |
| rs11078713 | -0.0096075 | 0.41765 | 17 | 7795972 | 0.0022544 | 344104 | 2.03E-05 |
| rs1154693 | 0.0027199 | 0.85141 | 3 | 117804154 | 0.0031363 | 344104 | 0.38582 |
| rs1160685 | 0.0010831 | 0.44087 | 4 | 94052854 | 0.0022381 | 344104 | 0.62843 |
| rs11658881 | -0.0051925 | 0.42029 | 17 | 2072949 | 0.0022526 | 344104 | 0.0211602 |
| rs11712680 | -0.0052946 | 0.18622 | 3 | 75009019 | 0.0028525 | 344104 | 0.0634366 |
| rs117143374 | -0.0095734 | 0.14188 | 21 | 40555561 | 0.0031913 | 344104 | 0.00270147 |
| rs11872397 | -0.0028089 | 0.2598 | 18 | 72535282 | 0.0025622 | 344104 | 0.27295 |
| rs12025237 | -0.012892 | 0.12589 | 1 | 154205120 | 0.0033484 | 344104 | 0.00011801 |
| rs12042107 | -0.0061504 | 0.54985 | 1 | 91196176 | 0.0022364 | 344104 | 0.00595799 |
| rs12112638 | 0.0017675 | 0.26336 | 7 | 69735251 | 0.0025228 | 344104 | 0.48354 |
| rs12186738 | -0.0022098 | 0.14225 | 5 | 103816655 | 0.0031796 | 344104 | 0.487059 |
| rs12333760 | -0.013235 | 0.16502 | 7 | 99185406 | 0.0029962 | 344104 | 1.00E-05 |
| rs12356821 | 0.0032754 | 0.14865 | 10 | 104563808 | 0.0031638 | 344104 | 0.30055 |
| rs12441907 | 0.0034208 | 0.18912 | 15 | 83922387 | 0.0028603 | 344104 | 0.23172 |
| rs12474587 | -0.0017175 | 0.45077 | 2 | 162802993 | 0.0022356 | 344104 | 0.44235 |
| rs12545053 | -0.0035226 | 0.40325 | 8 | 65073605 | 0.0022692 | 344104 | 0.12058 |
| rs12632110 | -0.0018212 | 0.65822 | 3 | 50224225 | 0.0023461 | 344104 | 0.43759 |
| rs13030994 | 0.0019845 | 0.4823 | 2 | 146143090 | 0.0022223 | 344104 | 0.37187 |
| rs13145728 | -0.0077389 | 0.38205 | 4 | 140927812 | 0.0022878 | 344104 | 0.00071796 |
| rs13261666 | -0.0082118 | 0.50399 | 8 | 59814666 | 0.0022202 | 344104 | 0.00021674 |
| rs134529 | -0.018144 | 0.38239 | 22 | 28781758 | 0.0022875 | 344104 | 2.17E-15 |
| rs1385108 | 0.0033267 | 0.24247 | 5 | 154839646 | 0.0025987 | 344104 | 0.2005 |
| rs1435741 | 0.0060151 | 0.42999 | 15 | 47935843 | 0.0022444 | 344104 | 0.00736224 |
| rs1445649 | 4.03E-05 | 0.53785 | 2 | 155682556 | 0.0022276 | 344104 | 0.98556 |
| rs1555445 | 0.010225 | 0.30931 | 20 | 31175258 | 0.0024128 | 344104 | 2.26E-05 |
| rs1565735 | -0.00065046 | 0.20142 | 8 | 27426077 | 0.0027879 | 344104 | 0.81551 |
| rs1869243 | 0.0025275 | 0.4698 | 3 | 5724536 | 0.0022311 | 344104 | 0.25727 |
| rs1899896 | 0.0088363 | 0.29753 | 8 | 93201036 | 0.0024361 | 344104 | 0.00028653 |
| rs1971318 | 0.060972 | 0.15853 | 12 | 121389500 | 0.0030438 | 344104 | 3.30E-89 |
| rs2046850 | -0.0029595 | 0.19443 | 1 | 210304319 | 0.0028158 | 344104 | 0.29325 |
| rs2050586 | 0.0014217 | 0.36008 | 1 | 87905828 | 0.002322 | 344104 | 0.54035 |
| rs2107300 | -0.0041975 | 0.84275 | 2 | 200937901 | 0.0030638 | 344104 | 0.17068 |
| rs2140114 | -0.002939 | 0.53667 | 7 | 3407568 | 0.0022282 | 344104 | 0.18717 |
| rs2186122 | 0.0086342 | 0.5615 | 1 | 66470206 | 0.0022534 | 344104 | 0.000127309 |
| rs222449 | -0.0095814 | 0.80234 | 6 | 52916062 | 0.002793 | 344104 | 0.000602629 |
| rs2378662 | -0.0017454 | 0.54233 | 9 | 86707289 | 0.0022366 | 344104 | 0.43517 |
| rs240963 | -0.003847 | 0.84066 | 6 | 111644332 | 0.0030383 | 344104 | 0.20546 |
| rs2631024 | -0.0048864 | 0.74023 | 8 | 91995577 | 0.0025422 | 344104 | 0.0545871 |
| rs266047 | -0.00028334 | 0.53459 | 2 | 104088751 | 0.0022237 | 344104 | 0.89861 |
| rs3001723 | 0.0062016 | 0.29981 | 1 | 44037685 | 0.0024208 | 344104 | 0.0104141 |
| rs301805 | 0.0097283 | 0.58608 | 1 | 8481016 | 0.0022554 | 344104 | 1.61E-05 |
| rs35702515 | 0.0069211 | 0.23543 | 2 | 137542847 | 0.0026417 | 344104 | 0.00879468 |
| rs3800227 | 0.0076923 | 0.7443 | 6 | 108994161 | 0.0025522 | 344104 | 0.00257852 |
| rs3801289 | -0.0019256 | 0.33792 | 7 | 96638267 | 0.0023544 | 344104 | 0.41342 |
| rs3904512 | 0.0039332 | 0.45073 | 13 | 38357471 | 0.0022344 | 344104 | 0.0783592 |
| rs4044321 | -0.0063407 | 0.64011 | 5 | 166989513 | 0.002317 | 344104 | 0.00620883 |
| rs4236259 | 0.0029657 | 0.49091 | 7 | 1708080 | 0.0022436 | 344104 | 0.18622 |
| rs4352629 | -0.0033009 | 0.4567 | 5 | 87756821 | 0.0022314 | 344104 | 0.13906 |
| rs4523689 | -0.0029844 | 0.39258 | 11 | 7950797 | 0.0022705 | 344104 | 0.1887 |
| rs4543592 | 0.000532 | 0.47991 | 9 | 3014254 | 0.0022255 | 344104 | 0.81107 |
| rs4674993 | -0.0024685 | 0.19891 | 2 | 226332033 | 0.0027821 | 344104 | 0.37493 |
| rs4759228 | -0.012891 | 0.29703 | 12 | 56508409 | 0.0024357 | 344104 | 1.21E-07 |
| rs4781977 | -0.00010995 | 0.22601 | 16 | 17572674 | 0.0026824 | 344104 | 0.9673 |
| rs4785836 | 0.001688 | 0.37619 | 16 | 65604652 | 0.0022968 | 344104 | 0.46238 |
| rs578584 | 0.0078512 | 0.55836 | 2 | 45143175 | 0.0022394 | 344104 | 0.000454999 |
| rs6265 | -0.0063556 | 0.18869 | 11 | 27679916 | 0.0028363 | 344104 | 0.0250409 |
| rs6433897 | 0.005891 | 0.73637 | 2 | 182034448 | 0.0025232 | 344104 | 0.0195578 |
| rs6508144 | -0.0050177 | 0.56995 | 18 | 50026142 | 0.0022486 | 344104 | 0.0256478 |
| rs66680800 | -0.0061931 | 0.39963 | 3 | 85985324 | 0.0022655 | 344104 | 0.00626369 |
| rs6669839 | 0.0074667 | 0.20991 | 1 | 50625979 | 0.0027303 | 344104 | 0.00624367 |
| rs6728726 | 0.006844 | 0.82766 | 2 | 623976 | 0.0029356 | 344104 | 0.019736 |
| rs6788098 | 0.0010834 | 0.62822 | 3 | 85624131 | 0.0022953 | 344104 | 0.63693 |
| rs6893752 | -0.0013113 | 0.74088 | 5 | 60374912 | 0.0025353 | 344104 | 0.60499 |
| rs7197072 | 0.0011984 | 0.22628 | 16 | 717085 | 0.0026571 | 344104 | 0.65199 |
| rs7224742 | -0.0015942 | 0.61985 | 17 | 30657058 | 0.00229 | 344104 | 0.48635 |
| rs72789632 | 0.00022347 | 0.13049 | 5 | 106834363 | 0.0033157 | 344104 | 0.94627 |
| rs72896886 | -0.0053682 | 0.16738 | 18 | 42632652 | 0.0029855 | 344104 | 0.0721573 |
| rs7322872 | -0.0039451 | 0.79016 | 13 | 100548329 | 0.002736 | 344104 | 0.14933 |
| rs7555507 | 0.0011585 | 0.51466 | 1 | 73766037 | 0.0022241 | 344104 | 0.60245 |
| rs7585579 | -0.0047184 | 0.50973 | 2 | 60024857 | 0.0022434 | 344104 | 0.0354462 |
| rs76214862 | -0.0047949 | 0.1869 | 14 | 29500130 | 0.0028546 | 344104 | 0.0930122 |
| rs76608582 | 0.0032548 | 0.04762 | 19 | 4474725 | 0.0054727 | 344104 | 0.55202 |
| rs7921378 | -0.0031892 | 0.48091 | 10 | 63674885 | 0.0022302 | 344104 | 0.15272 |
| rs7929518 | 0.0050589 | 0.78124 | 11 | 85980958 | 0.0026858 | 344104 | 0.0596266 |
| rs7938812 | 0.0035496 | 0.38675 | 11 | 112911004 | 0.0022823 | 344104 | 0.11988 |
| rs7969559 | -0.0085624 | 0.72267 | 12 | 69655167 | 0.0024806 | 344104 | 0.000557186 |
| rs9401770 | -0.0012187 | 0.26858 | 6 | 98748008 | 0.0025086 | 344104 | 0.62711 |
| rs9423279 | 0.0022829 | 0.65675 | 10 | 125680419 | 0.0023824 | 344104 | 0.33794 |
| rs9540729 | -0.0068987 | 0.52225 | 13 | 66947124 | 0.0022227 | 344104 | 0.00191069 |
| rs962625 | 0.0044478 | 0.26859 | 4 | 28473524 | 0.0025148 | 344104 | 0.0769502 |
| rs9835772 | -1.04E-05 | 0.24267 | 3 | 85766025 | 0.0025891 | 344104 | 0.9968 |
| rs993700 | -0.0077143 | 0.77773 | 4 | 67825894 | 0.0026703 | 344104 | 0.00386554 |

**Supplementary Table 17.** Detailed information on the SNPs for the MR analysis of smoking initiation and total protein(TP).

Removing the following SNPs for being palindromic with intermediate allele frequencies:

rs1160685, rs2186122, rs578584, rs6508144, rs7585579, rs7921378, rs9540729

| **SNP** | **beta.outcome** | **eaf.outcome** | | **chr** | **pos** | **se.outcome** | **samplesize.outcome** | **pval.outcome** |
| --- | --- | --- | --- | --- | --- | --- | --- | --- |
| rs10001365 | -0.0014793 | | 0.38611 | 4 | 147797214 | 0.0025806 | 314921 | 0.5665 |
| rs10114490 | -0.0021011 | | 0.1857 | 9 | 11070165 | 0.0032292 | 314921 | 0.51527 |
| rs10159545 | -0.0073709 | | 0.34787 | 10 | 21766969 | 0.0026594 | 314921 | 0.00557763 |
| rs10233018 | 0.0014658 | | 0.50399 | 7 | 117523709 | 0.0025158 | 314921 | 0.560141 |
| rs10260968 | 0.001155 | | 0.58905 | 7 | 1889773 | 0.0025517 | 314921 | 0.65081 |
| rs10279261 | -0.0015872 | | 0.61778 | 7 | 133589846 | 0.002596 | 314921 | 0.54092 |
| rs10498846 | -0.00041378 | | 0.5073 | 6 | 67405337 | 0.0025279 | 314921 | 0.86998 |
| rs1050847 | 0.009343 | | 0.57915 | 16 | 87443734 | 0.0025518 | 314921 | 0.000250981 |
| rs10905461 | 0.00023281 | | 0.74403 | 10 | 8803551 | 0.0028812 | 314921 | 0.9356 |
| rs11057005 | -0.0012246 | | 0.43763 | 12 | 16748721 | 0.0025486 | 314921 | 0.63088 |
| rs11078713 | 0.009971 | | 0.41771 | 17 | 7795972 | 0.0025507 | 314921 | 9.26E-05 |
| rs1154693 | -0.00298 | | 0.85127 | 3 | 117804154 | 0.0035473 | 314921 | 0.40087 |
| rs1160685 | -0.002461 | | 0.44086 | 4 | 94052854 | 0.0025321 | 314921 | 0.33108 |
| rs11658881 | -0.014365 | | 0.42026 | 17 | 2072949 | 0.0025494 | 314921 | 1.76E-08 |
| rs11712680 | 0.0046364 | | 0.18626 | 3 | 75009019 | 0.0032271 | 314921 | 0.15081 |
| rs117143374 | -0.0013175 | | 0.14193 | 21 | 40555561 | 0.0036094 | 314921 | 0.7151 |
| rs11872397 | 0.0039625 | | 0.2598 | 18 | 72535282 | 0.0029001 | 314921 | 0.17183 |
| rs12025237 | 0.0033076 | | 0.12569 | 1 | 154205120 | 0.003793 | 314921 | 0.38319 |
| rs12042107 | 0.0012041 | | 0.54962 | 1 | 91196176 | 0.0025298 | 314921 | 0.6341 |
| rs12112638 | 0.0026489 | | 0.26325 | 7 | 69735251 | 0.0028555 | 314921 | 0.35359 |
| rs12186738 | -0.00019533 | | 0.14234 | 5 | 103816655 | 0.0035972 | 314921 | 0.9567 |
| rs12333760 | 0.0077328 | | 0.16508 | 7 | 99185406 | 0.0033905 | 314921 | 0.0225642 |
| rs12356821 | 7.56E-05 | | 0.14871 | 10 | 104563808 | 0.0035796 | 314921 | 0.98315 |
| rs12441907 | -0.0032499 | | 0.1891 | 15 | 83922387 | 0.0032362 | 314921 | 0.31527 |
| rs12474587 | 0.0022851 | | 0.45087 | 2 | 162802993 | 0.0025299 | 314921 | 0.36639 |
| rs12545053 | 0.00115 | | 0.40341 | 8 | 65073605 | 0.0025682 | 314921 | 0.6543 |
| rs12632110 | -0.014146 | | 0.6579 | 3 | 50224225 | 0.002655 | 314921 | 9.94E-08 |
| rs13030994 | -0.0052712 | | 0.48222 | 2 | 146143090 | 0.0025148 | 314921 | 0.0360753 |
| rs13145728 | -0.00256 | | 0.38208 | 4 | 140927812 | 0.0025884 | 314921 | 0.32265 |
| rs13261666 | -0.0018675 | | 0.50376 | 8 | 59814666 | 0.0025133 | 314921 | 0.45746 |
| rs134529 | 0.001013 | | 0.38226 | 22 | 28781758 | 0.0025889 | 314921 | 0.69557 |
| rs1385108 | 0.0027004 | | 0.24248 | 5 | 154839646 | 0.0029408 | 314921 | 0.35848 |
| rs1435741 | 0.0040353 | | 0.42981 | 15 | 47935843 | 0.0025392 | 314921 | 0.11202 |
| rs1445649 | -0.0053426 | | 0.53766 | 2 | 155682556 | 0.0025198 | 314921 | 0.0339836 |
| rs1555445 | -0.007612 | | 0.30942 | 20 | 31175258 | 0.0027298 | 314921 | 0.0052959 |
| rs1565735 | 0.005724 | | 0.20161 | 8 | 27426077 | 0.0031527 | 314921 | 0.0694337 |
| rs1869243 | 0.0045191 | | 0.46956 | 3 | 5724536 | 0.0025241 | 314921 | 0.0733888 |
| rs1899896 | 0.0019741 | | 0.29754 | 8 | 93201036 | 0.0027571 | 314921 | 0.474 |
| rs1971318 | -0.014427 | | 0.15858 | 12 | 121389500 | 0.0034465 | 314921 | 2.84E-05 |
| rs2046850 | 0.0011223 | | 0.19454 | 1 | 210304319 | 0.0031851 | 314921 | 0.724559 |
| rs2050586 | 0.0050278 | | 0.3601 | 1 | 87905828 | 0.0026268 | 314921 | 0.0556186 |
| rs2107300 | -0.0025609 | | 0.84272 | 2 | 200937901 | 0.003467 | 314921 | 0.46013 |
| rs2140114 | -0.0007152 | | 0.53685 | 7 | 3407568 | 0.0025216 | 314921 | 0.776691 |
| rs2186122 | -0.0030595 | | 0.56147 | 1 | 66470206 | 0.00255 | 314921 | 0.23021 |
| rs222449 | -0.0018236 | | 0.80207 | 6 | 52916062 | 0.0031594 | 314921 | 0.56381 |
| rs2378662 | -0.002361 | | 0.54228 | 9 | 86707289 | 0.0025308 | 314921 | 0.35087 |
| rs240963 | 0.0055651 | | 0.84058 | 6 | 111644332 | 0.0034384 | 314921 | 0.10555 |
| rs2631024 | -0.0043134 | | 0.74016 | 8 | 91995577 | 0.0028772 | 314921 | 0.13383 |
| rs266047 | 0.0034315 | | 0.53452 | 2 | 104088751 | 0.0025166 | 314921 | 0.1727 |
| rs3001723 | -0.00398 | | 0.2998 | 1 | 44037685 | 0.0027393 | 314921 | 0.14625 |
| rs301805 | 0.011259 | | 0.58609 | 1 | 8481016 | 0.0025527 | 314921 | 1.03E-05 |
| rs35702515 | -0.00533 | | 0.23554 | 2 | 137542847 | 0.0029891 | 314921 | 0.0745624 |
| rs3800227 | -0.0041078 | | 0.74411 | 6 | 108994161 | 0.0028861 | 314921 | 0.15464 |
| rs3801289 | 0.0053065 | | 0.33782 | 7 | 96638267 | 0.0026638 | 314921 | 0.0463618 |
| rs3904512 | 0.00029464 | | 0.45059 | 13 | 38357471 | 0.0025284 | 314921 | 0.90723 |
| rs4044321 | -3.65E-05 | | 0.64042 | 5 | 166989513 | 0.0026235 | 314921 | 0.98889 |
| rs4236259 | -0.0019382 | | 0.49069 | 7 | 1708080 | 0.002539 | 314921 | 0.44524 |
| rs4352629 | -0.0023902 | | 0.45698 | 5 | 87756821 | 0.0025253 | 314921 | 0.34389 |
| rs4523689 | -0.0011329 | | 0.39269 | 11 | 7950797 | 0.0025689 | 314921 | 0.6592 |
| rs4543592 | 0.0022713 | | 0.47968 | 9 | 3014254 | 0.002519 | 314921 | 0.36725 |
| rs4674993 | 0.0017609 | | 0.19888 | 2 | 226332033 | 0.0031489 | 314921 | 0.576021 |
| rs4759228 | -0.0023022 | | 0.29709 | 12 | 56508409 | 0.0027569 | 314921 | 0.40367 |
| rs4781977 | 0.0049879 | | 0.22615 | 16 | 17572674 | 0.0030338 | 314921 | 0.10016 |
| rs4785836 | 0.0010765 | | 0.37619 | 16 | 65604652 | 0.002599 | 314921 | 0.678739 |
| rs578584 | 0.0016448 | | 0.55861 | 2 | 45143175 | 0.0025344 | 314921 | 0.51634 |
| rs6265 | 0.00283 | | 0.1887 | 11 | 27679916 | 0.0032102 | 314921 | 0.37802 |
| rs6433897 | -0.0060826 | | 0.73644 | 2 | 182034448 | 0.0028554 | 314921 | 0.0331581 |
| rs6508144 | -0.0022346 | | 0.56974 | 18 | 50026142 | 0.0025439 | 314921 | 0.37971 |
| rs66680800 | -0.0050102 | | 0.39985 | 3 | 85985324 | 0.0025636 | 314921 | 0.0506617 |
| rs6669839 | 2.70E-05 | | 0.2097 | 1 | 50625979 | 0.003091 | 314921 | 0.99302 |
| rs6728726 | 0.0024596 | | 0.82775 | 2 | 623976 | 0.0033216 | 314921 | 0.459 |
| rs6788098 | 0.0054956 | | 0.62864 | 3 | 85624131 | 0.0025975 | 314921 | 0.0343692 |
| rs6893752 | 0.003653 | | 0.74092 | 5 | 60374912 | 0.0028688 | 314921 | 0.2029 |
| rs7197072 | -0.00097872 | | 0.22618 | 16 | 717085 | 0.0030082 | 314921 | 0.744921 |
| rs7224742 | -0.0065752 | | 0.61975 | 17 | 30657058 | 0.0025909 | 314921 | 0.0111571 |
| rs72789632 | -0.00038933 | | 0.13052 | 5 | 106834363 | 0.0037518 | 314921 | 0.91735 |
| rs72896886 | -0.0075963 | | 0.16731 | 18 | 42632652 | 0.0033786 | 314921 | 0.024555 |
| rs7322872 | 0.0015707 | | 0.79026 | 13 | 100548329 | 0.0030958 | 314921 | 0.611911 |
| rs7555507 | 0.0037593 | | 0.51468 | 1 | 73766037 | 0.002517 | 314921 | 0.13529 |
| rs7585579 | 0.001379 | | 0.50995 | 2 | 60024857 | 0.0025395 | 314921 | 0.58713 |
| rs76214862 | 0.0017714 | | 0.18672 | 14 | 29500130 | 0.0032314 | 314921 | 0.583561 |
| rs76608582 | -0.0013488 | | 0.047557 | 19 | 4474725 | 0.0061999 | 314921 | 0.82778 |
| rs7921378 | 0.0080658 | | 0.48073 | 10 | 63674885 | 0.0025232 | 314921 | 0.00139059 |
| rs7929518 | -0.0021147 | | 0.78114 | 11 | 85980958 | 0.0030381 | 314921 | 0.48639 |
| rs7938812 | -0.0011798 | | 0.38707 | 11 | 112911004 | 0.0025824 | 314921 | 0.647781 |
| rs7969559 | -0.0063458 | | 0.72281 | 12 | 69655167 | 0.0028072 | 314921 | 0.0237881 |
| rs9401770 | -0.010887 | | 0.26879 | 6 | 98748008 | 0.0028372 | 314921 | 0.000124509 |
| rs9423279 | 0.0039339 | | 0.65651 | 10 | 125680419 | 0.002696 | 314921 | 0.14453 |
| rs9540729 | 0.0014009 | | 0.52252 | 13 | 66947124 | 0.0025146 | 314921 | 0.57746 |
| rs962625 | 0.0013473 | | 0.26863 | 4 | 28473524 | 0.0028444 | 314921 | 0.63573 |
| rs9835772 | -0.006363 | | 0.24241 | 3 | 85766025 | 0.0029316 | 314921 | 0.029973 |
| rs993700 | -0.00039065 | | 0.77766 | 4 | 67825894 | 0.0030221 | 314921 | 0.89715 |

**Supplementary Table 18.** MR-Egger analysis for testing potential horizontal pleiotropy.

| **Outcome** | **Egger intercept** | ***P*-value** |
| --- | --- | --- |
| ALP | 0.000732307 | 0.8038929 |
| ALT | 0.003319109 | 0.1975326 |
| TBIL | 0.000528986 | 0.8143719 |
| ALB | -0.000330544 | 0.9056169 |
| GGT | 0.001806394 | 0.6372175 |
| AST | 0.002876517 | 0.23826 |
| TP | 0.001422611 | 0.6013034 |

**Supplementary Table 19.** Odds Ratios(ORs) with 95% confidence intervals(CIs) for the lung cancer risk models by backward stepwise logistics regression.

|  |
| --- |

|  |  |  |  |
| --- | --- | --- | --- |
| **Characteristic** | **OR** | **95% CI** | ***P*-value** |
| **ALB(g/L)** |  |  |  |
| <40 | 1(Ref) | — | — |
| 40-43 | 0.91 | 0.73, 1.15 | 0.4 |
| 43-46 | 0.75 | 0.60, 0.94 | 0.01 |
| 46-49 | 0.67 | 0.53, 0.86 | <0.001 |
| >49 | 0.59 | 0.43, 0.79 | <0.001 |
| **ALP(U/L)** |  |  |  |
| <55 | 1(Ref) | — | — |
| 55-75 | 1.14 | 0.92, 1.44 | 0.3 |
| 75-95 | 1.42 | 1.15, 1.78 | 0.002 |
| 95-115 | 1.91 | 1.54, 2.41 | <0.001 |
| >115 | 1.85 | 1.46, 2.35 | <0.001 |
| **ALT(U/L)** |  |  |  |
| <10 | 1(Ref) | — | — |
| 10-20 | 0.87 | 0.70, 1.09 | 0.2 |
| 20-30 | 0.67 | 0.53, 0.86 | 0.001 |
| 30-40 | 0.59 | 0.45, 0.78 | <0.001 |
| >40 | 0.54 | 0.38, 0.75 | <0.001 |
| **AST(U/L)** |  |  |  |
| <15 | 1(Ref) | — | — |
| 15-25 | 0.73 | 0.53, 1.02 | 0.052 |
| 25-35 | 0.66 | 0.48, 0.93 | 0.014 |
| 35-45 | 0.6 | 0.41, 0.89 | 0.01 |
| >45 | 0.84 | 0.46, 1.47 | 0.6 |
| **GGT(U/L)** |  |  |  |
| <15 | 0.78 | 0.64, 0.94 | 0.009 |
| 15-25 | 0.87 | 0.78, 0.98 | 0.017 |
| 25-35 | 1(Ref) | — | — |
| 35-45 | 1 | 0.87, 1.14 | >0.9 |
| >45 | 1.14 | 1.01, 1.28 | 0.028 |
| **TBIL(μmol/L)** |  |  |  |
| <5 | 1(Ref) | — | — |
| 5-7.5 | 0.93 | 0.81, 1.08 | 0.3 |
| 7.5-10 | 0.84 | 0.72, 0.97 | 0.021 |
| 10-12.5 | 0.81 | 0.68, 0.97 | 0.019 |
| >12.5 | 0.66 | 0.54, 0.80 | <0.001 |
| **TP(g/L)** |  |  |  |
| <62 | 1.38 | 0.75, 2.33 | 0.3 |
| 62-68 | 1.02 | 0.91, 1.15 | 0.7 |
| 68-74 | 1(Ref) | — | — |
| 74-80 | 1.09 | 0.98, 1.20 | 0.1 |
| >80 | 1.46 | 1.16, 1.81 | <0.001 |
| **Age(years)** | 1.08 | 1.08, 1.09 | <0.001 |
| **Sex** |  |  |  |
| Male | 1(Ref) | — | — |
| Female | 0.7 | 0.64, 0.78 | <0.001 |
| **FEV1(liters)** |  |  |  |
| <3 | 1(Ref) | — | — |
| 3~4 | 0.53 | 0.47, 0.60 | <0.001 |
| >4 | 0.28 | 0.19, 0.39 | <0.001 |
| Unkown | 1.11 | 0.99, 1.24 | 0.079 |
| **History of hay fever and/or allergic rhinitis and/or eczma** |  |  |  |
| No | 1(Ref) | — | — |
| Yes | 0.78 | 0.69, 0.87 | <0.001 |
| **Family history of lung cancer** |  |  |  |
| No | 1(Ref) | — | — |
| Yes | 1.49 | 1.34, 1.66 | <0.001 |
| Unkown | 1.16 | 0.95, 1.41 | 0.13 |
| **History of emphyse and/or machronic and/or bronchitis** |  |  |  |
| No | 1(Ref) | — | — |
| Yes | 2.45 | 2.12, 2.82 | <0.001 |
| **smoking-packyears** |  |  |  |
| <10 | 1(Ref) | — | — |
| 10-20 | 1.35 | 1.13, 1.62 | <0.001 |
| >20 | 4.99 | 4.23, 5.94 | <0.001 |
